# Dual STAT3/STAT5 inhibition as a novel treatment strategy in T-prolymphocytic leukemia

**DOI:** 10.1038/s41375-025-02577-8

**Published:** 2025-04-15

**Authors:** Annika Dechow, Sanna Timonen, Aleksandr Ianevski, Qu Jiang, Linus Wahnschaffe, Yayi Peng, Dennis Jungherz, Kerstin Becker, Heidi A. Neubauer, Susann Schönefeldt, Elvin de Araujo, Patrick Gunning, Roman Fleck, Alexandra Schrader, Michael Hallek, Natali Pflug, Richard Moriggl, Tero Aittokallio, Satu Mustjoki, Till Braun, Marco Herling

**Affiliations:** 1 Department I of Internal Medicine, Center for Integrated Oncology Aachen-Bonn-Cologne-Düsseldorf, University Hospital Cologne, Cologne, Germany; 2 Hematology Research Unit Helsinki, University of Helsinki and Helsinki University Hospital Comprehensive Cancer Center, Helsinki, Finland; 3 Institute for Molecular Medicine Finland (FIMM), HiLIFE, University of Helsinki, Helsinki, Finland; 4 Translational Immunology Research Program, University of Helsinki, Helsinki, Finland; 5 Department of Hematology, Cellular Therapy, Hemostaseology and Infectious Diseases, University Hospital Leipzig, University of Leipzig Medical Center and Comprehensive Cancer Center Central Germany (CCCG), Leipzig-, Jena, Germany; 6 Cologne Center for Genomics (CCG), University of Cologne, Faculty of Medicine and University Hospital Cologne, Cologne, Germany; 7 Department for Biological Sciences and Pathobiology, University of Veterinary Medicine, Vienna, Austria; 8 Centre for Medicinal Chemistry, University of Toronto at Mississauga, Mississauga, ON L5L 1C6 Canada; 9 Janpix, A Centessa Company, London, UK; 10 Lymphoma Immuno Biology Team, Equipe Labellisée LIGUE 2023, Centre International de Recherche en Infectiologie, INSERM U1111-CNRS UMR5308, Faculté de Médecine Lyon-Sud, Hospices Civils de Lyon, Université Claude Bernard Lyon I-ENS de Lyon, Lyon, France; 11 Department of Biosciences and Medical Biology, Paris Lodron University of Salzburg, Salzburg, Austria; 12 Institute for Cancer Research, Department of Cancer Genetics, Oslo University Hospital, Oslo, Norway; 13 iCAN Digital Precision Cancer Medicine Flagship, University of Helsinki and Helsinki University Hospital, Helsinki, Finland; 14 Department of Clinical Chemistry and Hematology, University of Helsinki, Helsinki, Finland; 15 Mildred Scheel School of Oncology Aachen Bonn Cologne Düsseldorf (MSSO ABCD), Cologne, Faculty of Medicine and University Hospital of Cologne, Cologne, Germany

**Keywords:** Targeted therapies, Translational research, Leukaemia

## Abstract

T-prolymphocytic leukemia (T-PLL) is a rare, aggressive T-cell malignancy with poor outcomes and an urgent need for new therapeutic approaches. Integrating genomic data and new transcriptomic profiling, we identified recurrent *JAK/STAT* mutations (predominantly in *JAK3* and *STAT5B*) as hallmarks in a cohort of 335 T-PLL cases. In line, transcriptomic and protein analyses revealed constitutive JAK/STAT activation in virtually all samples. Consequently, we explored the anti-leukemic potential of dual STAT3/STAT5 non-PROTAC degraders in T-PLL, with JPX-1244 as our lead substance. JPX-1244 efficiently and selectively induced cell death in primary T-PLL samples, including those resistant to conventional therapies, by blocking STAT3 and STAT5 phosphorylation and by inducing their degradation. The extent of STAT3/STAT5 degradation directly correlated with cytotoxicity. RNA-sequencing confirmed the treatment-related downregulation of STAT5 target genes. While *JAK/STAT* mutations did not predict responses to pharmacologic STAT3/STAT5 degradation, elevated expression of *TOX, PAK6*, and *SPINT1* were associated with drug sensitivity. In subsequent combination screenings, cladribine, venetoclax, and azacytidine emerged as most effective combination partners of STAT3/STAT5 degraders, even in low-responding T-PLL samples, all synergistically reducing STAT5 phosphorylation. These findings highlight dual STAT3/STAT5 inhibition, particularly in combination with hypomethylating and BCL2-targeting agents, as a promising interventional approach in T-PLL, warranting further investigation.

## Introduction

T-prolymphocytic leukemia (T-PLL) is a rare T-cell neoplasm with an incidence of ~2 cases per million, comprising ~2% of mature lymphocytic leukemias [1–3]. T-PLL patients typically present in their 7^th^ decade with rapidly rising lymphocyte counts, hematopoietic impairments due to bone marrow infiltration, splenomegaly, and small lymphadenopathy [1, 4–6]. Conventional chemotherapies show minimal efficacy [7]. The monoclonal CD52 antibody alemtuzumab induces remission in ~90% of therapy-naïve cases [8–10], although relapses are almost inevitable [7]. Long-term remissions can be achieved through consolidating allogeneic stem cell transplantation in the small subset of eligible patients [11]. This results in a low median overall survival of T-PLL patients of ≤3 years from diagnosis [12].

The most common molecular hallmarks in T-PLL pathogenesis involve rearrangements of the *T-cell leukemia 1* (TCL1) family gene loci of *TCL1A* or *MTCP1*. This juxtapositions them to T-cell receptor (TCR) gene enhancer elements, resulting in constitutive expression of these TCR signal amplifying oncogenes [5, 13, 14]. Loss of functional Ataxia Telangiectasia Mutated (ATM), due to deletions or mutations, impairs DNA damage check points and cooperates with TCL1A overexpression [15–17]. Subsequent hits of postulated leukemogenic importance involve MYC, AGO2, the miR-ome, and epigenetic regulators [15, 18, 19].

Recently, constitutive JAK/STAT signaling was uncovered as another central node in T-PLL, mainly elicited by recurrent genomic lesions, predominantly gain-of-function (GOF) mutations in *JAK1*, *JAK3*, and *STAT5B*, supplemented by genomic losses of negative JAK/STAT regulators such as *DUSP4* and *SHP1* [20]. The JAK/STAT pathway is a well-described membrane-to-nucleus signaling system with various effector functions. STAT3, STAT5A, and STAT5B act as transcription factors upon phosphorylation and nuclear transfer in response to JAK activation by cytokines or growth factors [21, 22]. Constitutive activation of STAT3/STAT5 enhances cell proliferation and prevents apoptosis, both linked to the leukemogenesis of various entities [23–25].

There is limited clinical evidence supporting the use of JAK inhibitors like ruxolitinib and tofacitinib in T-PLL [26–28], including a case demonstrating activity when combined with the BCL2 inhibitor venetoclax [29]. However, in more extensive ex-vivo screenings of primary T-PLL samples, we reported a lack of efficacy for JAK inhibitors [30, 31]. This implies that the effector molecules STAT3 and STAT5 represent a more specific vulnerability in T-PLL.

The therapeutic efficacy of single STAT5 inhibition in other entities was limited through compensatory STAT3 activation via a SOCS2-mediated feedback loop [32, 33], highlighting the need for dual STAT3/STAT5 inhibition to overcome STAT dependence and prevent bypass mechanisms. Although preclinical studies explored SH2 domain-targeting peptides, small molecules, and PROTAC-based strategies, no specific STAT3 or STAT5 inhibitor is clinically available, mainly due to issues of bioavailability, toxicity, and insufficient antitumor efficacy [34]. Novel compounds from the JPX-series irreversibly bind to cysteine residues in STAT proteins through nucleophilic aromatic substitution, inducing protein destabilization and degradation [35, 36]. These non-PROTAC small-molecule inhibitors showed encouraging results in cutaneous T-cell lymphoma (CTCL) [37], T-cell acute lymphoblastic leukemia (T-ALL) [38], and acute myeloid leukemia (AML) [35].

Here, we present a comprehensive analysis establishing constitutive JAK/STAT signaling as a key driver in T-PLL, supported by a meta-analysis of 335 cases. We demonstrate the therapeutic potential of dual STAT3/STAT5 non-PROTAC degraders from the JPX-series, which efficiently inhibit STAT phosphorylation and protein stability, correlating with ex-vivo treatment responses and significantly altered STAT target genes. Combination strategies with agents like cladribine, azacytidine, and venetoclax further enhance efficacy, even in low-responding cases, highlighting dual STAT3/STAT5 inhibition as a promising therapeutic approach in T-PLL.

## Materials and methods

### Meta-analyses of genomic JAK/STAT aberrations

We combined 275 T-PLL cases previously published in our meta-analysis [20] with 60 newly characterized cases (*n* = 30 newly sequenced; *n* = 30 re-analyzed [39]), resulting in 335 cases with sequencing data on at least one *JAK* or *STAT* gene locus, including 147 cases with available sequencing data for all *JAK/STAT* genes. The distribution of cases and sequencing methods are in Supplementary Table 1.

### Patient cohort

We included peripheral blood-derived samples of 58 T-PLL patients and 16 age-matched (>55 years) healthy donors in ex-vivo experiments (patient characteristics in Supplementary Table 2). The diagnosis was confirmed according to WHO criteria and consensus guidelines [1]. All patients provided informed consent. Collection and use of samples for research purposes were approved by the Ethics Committee of the Universities of Cologne (#12-146, #19-089) and Helsinki (303/13/03/01/2011).

### Single-compound and combination drug testing

The single-drug and combination screening included 28 JPX compounds, 19 combination partners, and bendamustine, cytarabine and ruxolitinib as comparator substances. In these screenings, we either assessed cell viability using the CellTiter-Glo (CTG) luminescent assay (Promega; single-agent screening of JPX-series and combination screening, evaluating IC50), or cell death via AnnexinV-APC/7AAD flow cytometry (#640941, #420404, both BioLegend; all other experiments, evaluating LD50), according to standard protocols.

### RNA-sequencing

In total, 118 samples were sequenced, comprising 32 primary T-PLL samples without treatment and/or stimulation, and 11 T-PLL patients in the context of JPX-1244 treatment (compared to DMSO control) and/or IL-6 stimulation (compared to unstimulated control), resulting in 4 conditions per patient and timepoint (8 h and 24 h, *n* = 86 samples after exclusion of 2 samples with low RNA quality).

Further detailed information on the methodology of the meta-analysis, cell isolation and culturing, immunoblots, assessment of single-compound and combination screening data, RNA preparation, and processing of RNA-sequencing data are provided in the Supplementary Methods.

## Results

### Mutational landscape and activation state implicate enhanced JAK/STAT signaling as a central mechanism of T-PLL’s pathogenesis

**Fig. 1:**
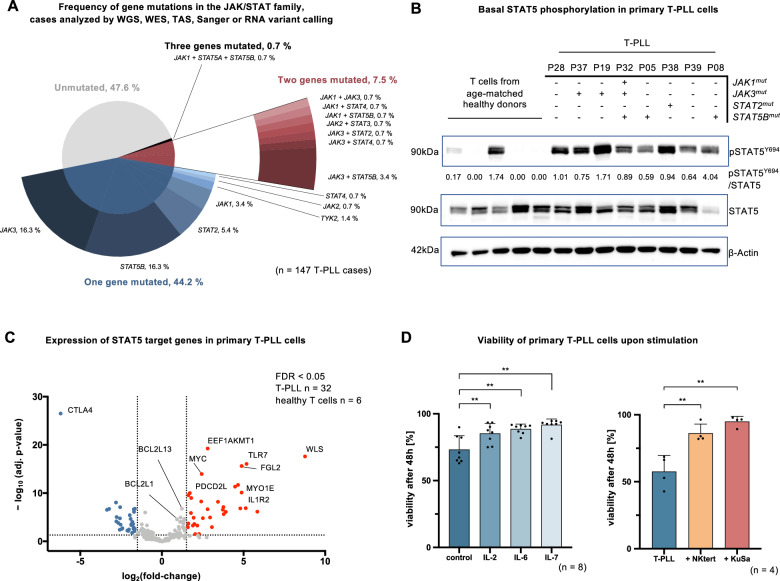
Genomic, transcriptomic, and proteomic signatures of enhanced JAK/STAT signaling in T-PLL. We expanded a previously published meta-analysis containing sequencing data on 275 T-PLL cases [20] with 60 additional cases, assessed for JAK/STAT mutations by RNA profiling, resulting in a cohort of 335 T-PLL patients with sequencing data on at least one *JAK* or *STAT* gene locus. **A** Frequency of *JAK* and *STAT* mutations in 147 T-PLL patients. Only patients with data on the mutational landscape of all JAK/STAT members were included. The inner pie chart displays the proportion of T-PLL patients carrying one (blue), two (claret), three (black), or no (gray) mutation in members of the JAK/STAT signaling pathway. The outer pie chart gives information on the affected *JAK/STAT* gene. **B** Basal phosphorylation of STAT5 in 8 T-PLL cases compared to healthy-donor derived CD3^+^ T cells (*n* = 5), as assessed via immunoblot. The JAK/STAT mutation status is presented for all T-PLL patients. Quantification of STAT5 phosphorylation was performed in comparison to total STAT5 expression, and β-Actin was used as a housekeeper. **C** Volcano plot of STAT5 target genes, adopted from the HALLMARK IL2-STAT5 targets gene list, in 32 T-PLL cases compared to healthy-donor derived T-cell controls (*n* = 6). Genes significantly upregulated in T-PLL cells are presented in red, downregulated genes are marked in blue (FDR < 0.05, |log_2_FC|≧1.5). **D** Bar chart displaying the viability of primary T-PLL cells with and without continuous stimulation with interleukins (IL-2 5 ng/ml; IL-6 2 ng/ml; IL-7 5 ng/ml) or in co-culture with the feeder cell lines KuSa and NKtert. Cell death was measured after 48 h via AnnexinV/7AAD flow cytometry (mean with SD is presented, two-tailed paired Student’s *t* test, **p < 0.01).

Building on our meta-analysis on JAK/STAT aberrations published in 2019 [20], we merged the previously reported set of 275 T-PLL with 60 additional cases. We assessed for *JAK/STAT* mutations by RNA profiling, ending up at a cohort of 335 well-characterized T-PLL (see Supplementary Table 1 for sources of cases and sequencing methods). We first sought to determine the relative frequencies of mutations in any *JAK* or *STAT* gene by only considering cases with full data available on all *JAK* or *STAT* genes (*n* = 147). In this cohort, 52.4% of patients (*n* = 77) carried at least one mutation in any *JAK* or *STAT* gene (Fig. 1A). Mutations in *JAK3* and *STAT5B* were most common with 16.3% each (*n* = 24), followed by *STAT2* with 5.4% (*n* = 8) and *JAK1* with 3.4% (*n* = 5). Co-occurring mutations were detected in 11 cases (7.5%), with *JAK3* plus *STAT5B* being the most frequent combination (3.4%, *n* = 5). One case (0.7%) exhibited three co-occurring mutations (*JAK1*, *STAT5A*, *STAT5B*).

Furthermore, we incorporated all T-PLL cases with sequencing data on each respective *JAK/STAT* gene locus, and observed even higher mutation rates, with 38.0% of cases being mutated in *JAK3* (*n* = 126/332), 21.2% in *STAT5B* (*n* = 57/269), 5.9% in *JAK1* (18/306), and 8.5% in *STAT2* (*n* = 12/142, Supplementary Fig. 1A). Mutations in *JAK1* and *JAK3* predominantly clustered in the negative regulatory Ser/Thr kinase domain (‘pseudokinase domain”) with the *V658F* missense mutation being the most frequent variant in *JAK1* (50.0%, *n* = 9/18, Supplementary Fig. 1B), indicative of hyperactivation of *JAK1*, and the *M511I* GOF missense mutation as the most frequent in *JAK3* (58.7%, *n* = 74/126) [40]. In *STAT2*, the most common missense mutation *Q815H* was detected in the C-terminal region (33.3%, *n* = 4/12). In *STAT5B*, virtually all mutations were found in the SH2 domain, with the *N642H* missense mutation, a GOF variant hyperactivating STAT5B by prolonged and enhanced tyrosine phosphorylation [41], as the most common (47.4%, *n* = 27/57).

We next investigated transcriptomic and proteomic signatures of JAK/STAT activation in T-PLL. As T-PLL shows a heterogeneous coreceptor spectrum that includes CD4^+^, CD8^+^, and CD4^+^CD8^+^ subsets (Supplementary Table 2), we utilized CD3^+^ pan-T cells derived from age-matched healthy donors as controls, capturing this respective diversity of T-PLL [42]. Remarkably, we found constitutively enhanced basal (unstimulated) phosphorylation of STAT5 in all investigated cases (*n* = 8), when compared to CD3^+^ T cells from healthy donors (*n* = 5). This was irrespective of *JAK/STAT* mutations (Fig. 1B), and suggests alternative modes of JAK/STAT activation besides GOF mutations. To further substantiate constitutive STAT5 activation, we analyzed the expression of STAT5 target genes in T-PLL cases (*n* = 32) in comparison to CD3^+^ T cells from age-matched healthy donors (*n* = 6) via bulk RNA-sequencing. Overall, 63 described STAT5 target genes were significantly differentially expressed (false discovery rate (FDR) < 0.05, fold change |log_2_FC|≧1.5), with the majority of differentially expressed genes (DEG) being significantly upregulated in T-PLL cells (e.g. *MYC* and *BCL2L1*; Fig. 1C). This constitutive activation, independent of GOF mutations, is in line with previously reported genomic losses of negative regulators of JAK/STAT signaling in T-PLL [20]. To consolidate these findings, we assessed gene expression levels of key regulators (Supplementary Table 3) in 32 T-PLL cases (Supplementary Fig. 1C). Fittingly, we observed a significant downregulation of negative regulators, e.g. *DUSP2* and *PTPN13*, and an upregulation of positive regulators of JAK/STAT signaling, e.g. *IL5RA* and *IL6R*, in T-PLL, as compared to healthy-donor CD3^+^ T cells (*n* = 6). Next, we aimed to investigate how extrinsic cytokine stimulation, predominantly mediating signals via the JAK/STAT pathway, impacts the viability of T-PLL samples. For this, T-PLL cells were exposed to IL-2, IL-6, or IL-7 (*n* = 8), or co-cultured with the bone marrow stromal feeder cell lines NKtert and KuSa (*n* = 4, Fig. 1D). Both models of micromilieu-derived stimulation significantly increased the viability of T-PLL cells, measured after 48 h incubation (control to IL-2 *p* = 0.0012, IL-6 *p* = 0.0018, IL-7 *p* = 0.0021, all Student’s *t* test), indicating added beneficial effects of exogenous JAK/STAT trigger despite the proven basal activation. These findings were supported by a gene set enrichment analysis (GSEA), which compared the RNA expression of genes in T-PLL cases (*n* = 32) to healthy-donor controls (*n* = 6), emphasizing increased cytokine signaling in T-PLL cells (Supplementary Fig. 1D).

### High ex-vivo potency and selectivity of the non-PROTAC dual STAT3/STAT5 inhibitor JPX-1244

**Fig. 2:**
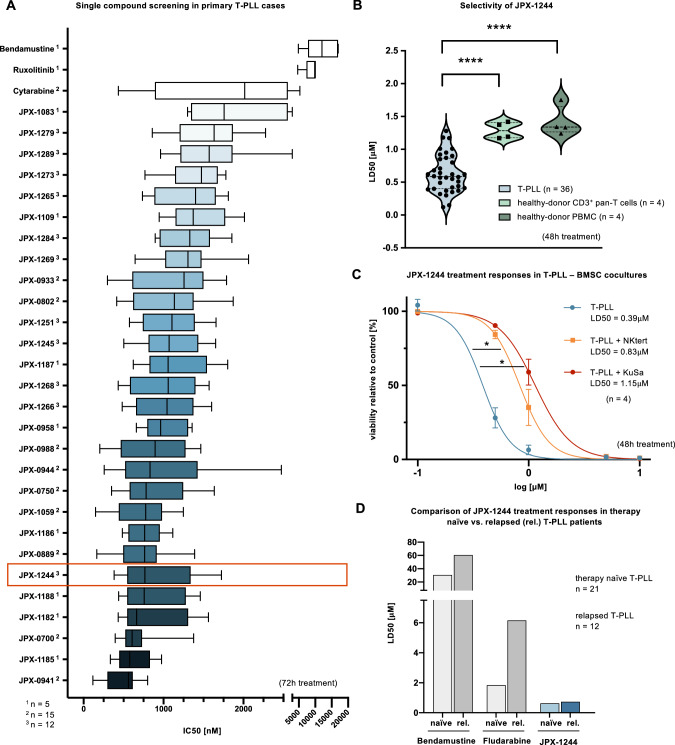
Dual STAT3/STAT5 inhibitors efficiently and selectively induce cell death in primary T-PLL cells ex-vivo. **A** Box plot showing inhibitory concentrations 50 (IC50s) of 28 dual Non-PROTAC STAT3/STAT5 degrader in primary T-PLL cases, in comparison to the clinically used cytostatics bendamustine and cytarabine, and the JAK inhibitor ruxolitinib. The number of T-PLL cases tested is given for each compound (^1^ *n* = 5, ^2^ *n* = 15, ^3^ *n* = 12). Mean with whiskers minimum to maximum are presented and substances were ordered from lowest to highest IC50. Cell viability was determined via cell titer glow (CTG) assay after 72 h treatment. JPX-1244 is marked as the selected lead substance, based on stability and safety data given in Supplementary Fig. 2A–C. **B** Violin plot of JPX-1244 lethal dosages 50 (LD50) values of 36 T-PLL cases, compared to CD3^+^ T cells and PBMC derived from age-matched healthy donors (*n* = 4). Cell death was determined by AnnexinV/7AAD flow cytometry after 48 h treatment with increasing concentrations of JPX-1244. The median is presented as an interrupted line and the quartiles as a dotted line. A two-tailed unpaired Student’s *t* test was performed (****p < 0.0001). **C** Dose-viability curves of primary T-PLL cells in monoculture or cocultures with stromal bone marrow feeder cell lines NKtert and KuSa (*n* = 4) upon treatment with increasing concentrations (0.1 µM to 10 µM) of JPX-1244 for 48 h (mean with SEM, two-way ANOVA with Geisser-Greenhouse correction and Bonferroni’s multiple comparisons test, *p < 0.05). **D** Bar chart displaying median LD50s of bendamustine, fludarabine, and JPX-1244 in 21 therapy naïve T-PLL cases compared to 12 relapsed (rel.) T-PLL patients. Median LD50s were calculated based on the median dose-viability curves after 48 h treatment with increasing concentrations of each compound, viability was assessed via AnnexinV/7AAD flow cytometry (see Supplementary Fig. 2E for individual dose-viability curves). Not all patients individually reached an LD50 (not reached with fludarabine: naïve 5/21 patients, relapsed 5/12 patients; not reached with bendamustine: naïve 13/21 patients, relapsed 5/12 patients with higher concentrations tested). The LD50 of bendamustine exhibited a 1.98-fold increase in relapsed patients, of fludarabine a 3.35 increase, and of JPX-1244 a 1.14-fold elevation, compared to treatment-naïve T-PLL cases.

Given the corroborated central role of basal and induced JAK/STAT signaling in T-PLL, we explored the potential of novel non-PROTAC small-molecule inhibitors from the JPX-series designed to simultaneously target STAT3 and STAT5. First, we conducted a single compound screening of 28 STAT3/STAT5 inhibitors in 5, 12, or 15 T-PLL cases (Fig. 2A). All 28 compounds efficiently reduced T-PLL cell viability in each case after 72 h treatment as determined by CTG luminescent assays, with IC50 values ranging from a mean of 0.49 µM (JPX-0941) to 1.91 µM (JPX-1083), which was lower than for the JAK1/JAK2 inhibitor ruxolitinib (IC50 = 8.93 µM) and for the cytostatic agents bendamustine (IC50 = 12.3 µM) or cytarabine (IC50 = 2.12 µM). We selected JPX-1244 as our lead substance for further experiments after evaluating stability and safety data ex vivo and in vivo. JPX-1244 demonstrated robust stability in whole blood and satisfactory glutathione stability alongside favorable plasma concentrations following intravenous or intraperitoneal administration in mice (Supplementary Fig. 2A–C).

Next, we investigated the efficacy and selectivity of JPX-1244 in a larger T-PLL cohort. The LD50 in 36 primary T-PLL cases (mean = 0.63 µM) was significantly lower than in healthy-donor CD3^+^ T cells (*n* = 4, mean = 1.29 µM, *p* < 0.001, Student’s *t* test) and in healthy-donor peripheral blood mononuclear cells (PBMCs, *n* = 4, mean = 1.42 µM, *p* < 0.0001, Student’s *t* test), as assessed by AnnexinV/7AAD flow cytometry after 48 h of treatment, implicating a selective anti-leukemic effect of JPX-1244 in T-PLL (Fig. 2B). We further validated the efficacy of JPX-1244 treatment in T-PLL-like cell lines, generated by introduction of TCL1A into T-cell leukemia lines (HuT78^TCL1A^, Jurkat^TCL1A^, HH^TCL1A^) and in SUP-T11 cells (carrying a t(14;14)). The comparable LD50s ranged from 0.48 µM to 0.84 µM (Supplementary Fig. 2D).

To investigate the efficacy of JPX-1244 in T-PLL in the context of simulated microenvironmental pro-survival stimulation, we performed cocultures of primary T-PLL cells with the bone marrow stromal cell (BMSC) lines NKtert and KuSa (*n* = 4, Fig. 2C). Although the LD50s of JPX-1244 increased in this setting (2.13-fold in NKtert condition, *p* = 0.0391; 2.95-fold with KuSa, *p* = 0.0166, two-way ANOVA), JPX-1244 overcame the feeder cell support in both cocultures. Furthermore, we investigated the activity of JPX-1244 in relapsed T-PLL (Fig. 2D), representing a serious clinical challenge in T-PLL therapy. Remarkably, the LD50 of JPX-1244 in relapsed cases (*n* = 12) was similar to those in samples from therapy-naïve T-PLL (*n* = 21, 1.14-fold increase), while bendamustine (1.98-fold increase) and fludarabine (3.35-fold increase), exhibited higher LD50 levels in these cases.

### JPX-1244-mediated STAT3/STAT5 dephosphorylation and degradation correlate with ex-vivo anti-leukemic responses

**Fig. 3:**
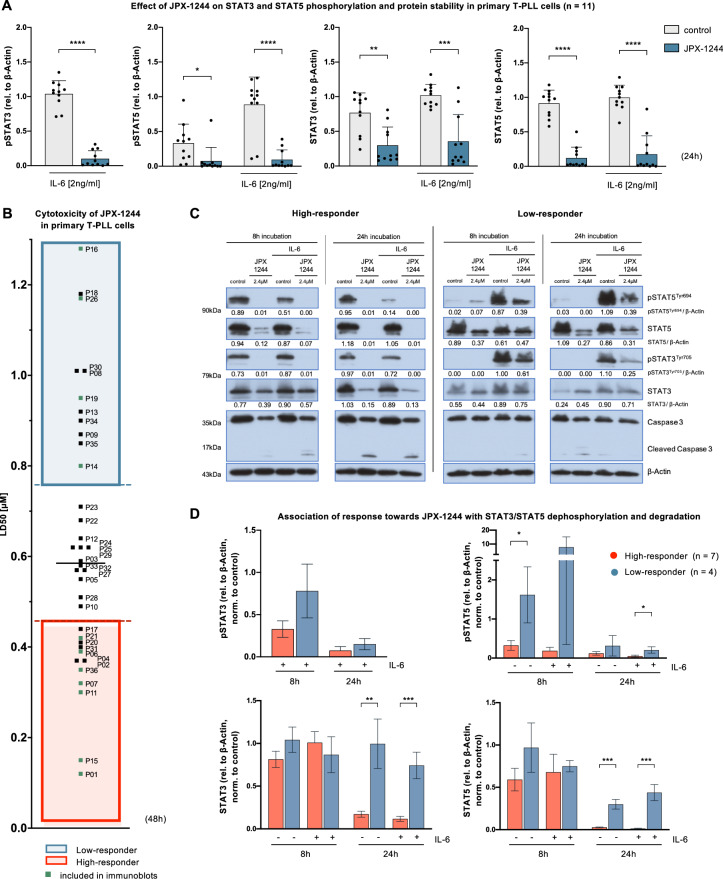
Underlying its marked anti-leukemic activity, JPX-1244 reduces STAT3/STAT5 phosphorylation paralleled by induced STAT3/STAT5 degradation. The effects of JPX-1244 treatment on STAT3 and STAT5 phosphorylation, as well as protein stability, were investigated in immunoblots of 11 T-PLL patients, treated with 2.4 µM JPX-1244 for 8 h or 24 h. To enhance the phosphorylation of STAT3 and STAT5 in patients with low basal phosphorylation levels, each condition was doubled and stimulation with IL-6 (2 ng/ml) was performed in one sample of each condition (see Supplementary Fig. 3A for pSTAT3 and pSTAT5 quantification upon IL-6 stimulation). **A** Quantification of densitometry of pSTAT3, pSTAT5, STAT3, and STAT5 signals, untreated or upon treatment with JPX-1244 for 24 h, assessed by immunoblots and normalized to β-Actin (mean with SD, two-tailed paired Student’s *t* test, *p < 0.05, **p < 0.01, ***p < 0.001, ****p < 0.0001). **B** Bar chart showing the distribution of LD50s upon JPX-1244 treatment in 36 T-PLL patients (0.12 µM to 1.28 µM), as determined in viability assays shown in Fig. 2B. Patients were divided in JPX-1244 High-responder (0.12–0.45 µM, *n* = 12) and Low-responder (0.75–1.3 µM, *n* = 11). T-PLL patients who have been assessed for STAT3/STAT5 phosphorylation and protein degradation upon JPX-1244 treatment are labeled in green (*n* = 11). **C** Exemplary immunoblots of one JPX-1244 High-responder (P11) and one Low-responder (P26) upon JPX-1244 treatment. Signals of pSTAT5^Tyr694^, STAT5, pSTAT3^Tyr705^, and STAT3 are shown, densitometric quantification of each was calculated relative to β-Actin. Cleavage of Caspase 3 signals is presented as apoptotic marker, and β-Actin was used as a housekeeper. **D** Quantification of pSTAT3, pSTAT5, STAT3, and STAT5 signals upon JPX-1244 treatment divided between low- (*n* = 4) and high-responding cases (*n* = 7). Densitometric assessments were calculated relative to β-Actin and normalized to the untreated control (mean with SEM, two-tailed unpaired Student’s *t* test, *p < 0.05, **p < 0.01, ***p < 0.001). Quantification of pSTAT3 signals was only performed upon IL-6 stimulation, as most of the T-PLL patients did not show basal STAT3 phosphorylation.

Having established the ex-vivo efficacy and selectivity of JPX-1244 in T-PLL, we explored the mode(s) of action of this inhibitor designed to target STAT3 and STAT5. We compared the phosphorylation and protein stability of STAT3 and STAT5 in 11 T-PLL cases after exposure to (sub-lethal) 2.4 µM JPX-1244 for 8 h and 24 h. We also stimulated one part of each condition with 2 ng/ml IL-6 to additionally induce phosphorylation of STAT3 and STAT5 in T-PLL cases with lower basal phosphorylation levels (Supplementary Fig. 3A, B). Viability of T-PLL cells was above 50% at the time of harvest (Supplementary Fig. 3C). Treatment with JPX-1244 for 24 h led to a significant reduction of the IL-6-mediated phosphorylation of STAT3 (*p* < 0.0001) as well as a significant decrease in the unstimulated and IL-6 triggered phosphorylation of STAT5 (*p* = 0.0127 and *p* < 0.0001, respectively, all Student’s *t* test, Fig. 3A). This inhibited STAT3/STAT5 phosphorylation was followed by significant STAT3 and STAT5 degradation in both the unstimulated and IL-6 stimulated conditions (STAT3: *p* = 0.0012 unstimulated, *p* = 0.0002 upon IL-6 stimulation; STAT5: both *p* < 0.0001, all Student’s *t* test). While the impairment of STAT3/STAT5 phosphorylation already ensued after 8 h, the effects of JPX-1244 treatment on protein stability were more pronounced at the 24 h timepoint (Supplementary Fig. 3D).

Although all 36 T-PLL cases showed responses to JPX-1244 in our 48 h cytotoxicity assays (Fig. 2B), we observed a sizable range in LD50 from 0.12 µM (P01) to 1.28 µM (P16, Fig. 3B). Based on this ~1-log distribution, we divided the cases into the subgroups of high-responders (HR, LD50 < 0.45 µM, *n* = 12) and low-responders (LR, LD50 > 0.75 µM, *n* = 11) with the mid-tercile of 13 cases excluded for further comparisons. To associate cytotoxicity of JPX-1244 treatment with the degree of reduced STAT3/STAT5 phosphorylation and protein degradation, we compared immunoblots upon JPX-1244 treatment in 7 HR and 4 LR cases (Fig. 3C, D). HR T-PLL patients tended to show lower levels of STAT5 phosphorylation upon JPX-1244 treatment, both in unstimulated T-PLL after 8 h (*p* = 0.0419) and in IL-6 stimulated samples after 24 h (*p* = 0.0499, both Student’s *t* test). Strikingly, the extent of protein degradation of both STAT3 and STAT5 was consistently higher in HR than in LR patients after 24 h of JPX-1244 treatment (STAT3: *p* = 0.0040 unstimulated, *p* = 0.0005 upon IL-6 stimulation; STAT5: *p* = 0.0001 unstimulated, *p* = 0.0002 upon IL-6 stimulation, all Student’s *t* test). These data demonstrate a strong association between the biochemical mechanism of the dual STAT3/STAT5 degrader and its cytotoxic effects.

### Marked transcriptomic alterations of JAK/STAT targets upon JPX-1244 treatment

**Fig. 4:**
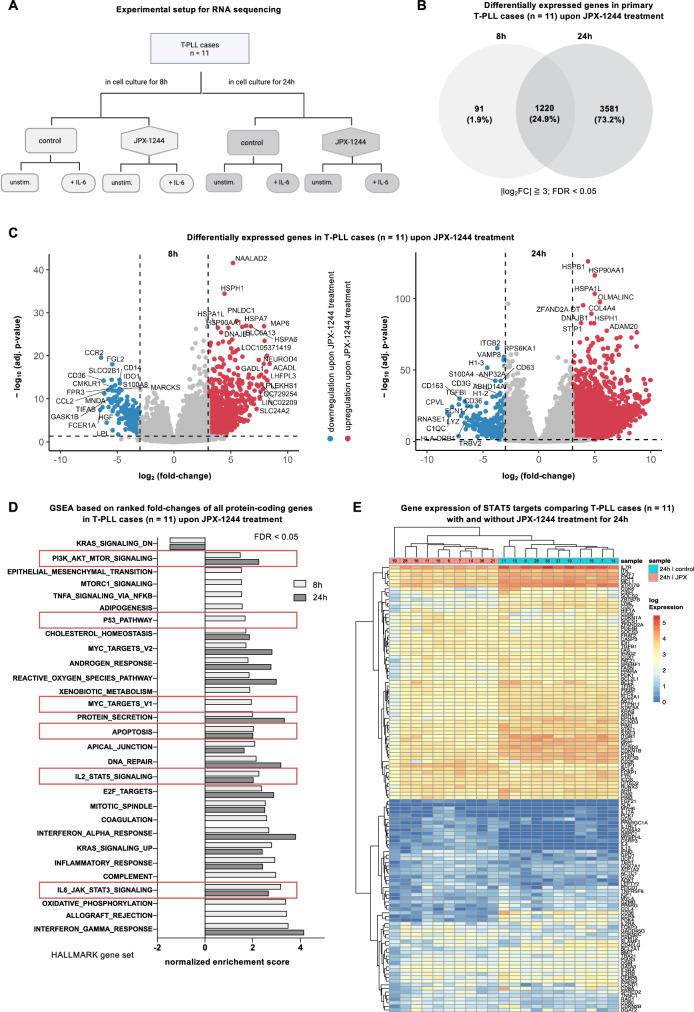
Marked transcriptomic alterations of JAK/STAT targets upon JPX-1244 exposure. **A** Flow chart visualizing the experimental setup for subsequent RNA-sequencing. In total, 11 T-PLL patients were treated, with 2.4 µM JPX-1244, either for 8 or 24 hours, against DMSO controls. In addition, in each condition, one sample was stimulated with 2 ng/ml IL-6, resulting in 8 samples per patient. **B** Venn diagram showing the overlap of differentially (|log_2_FC|≧3; FDR < 0.05) expressed genes (DEG) upon JPX-1244 treatment (without IL-6 stimulation) between 8 h and 24 h of treatment. While 91 genes were exclusively deregulated after 8 h (1.9%), 1,220 genes showed differential expression at both time points (24.9%), and 3581 genes (73.2%) were exclusively deregulated after 24 h of JPX-1244 treatment. **C** Volcano plot of DEGs upon JPX-1244 treatment (without IL-6 stimulation) after 8 h (left) and 24 h (right). Genes significantly downregulated upon JPX-1244 treatment are marked in blue, and genes upregulated upon JPX-1244 treatment are colored in red, with a cut-off at |log_2_FC|≧3 and an FDR < 0.05. **D** Gen set enrichment analyses (GSEAs) of HALLMARK gene sets upon JPX-1244 treatment compared to DMSO controls, presented for 8 h (light gray) and 24 h of treatment (dark gray). All significantly altered gene sets (FDR < 0.05) are presented in the graph. Gene sets of particular interest, specifically those related to apoptotic signaling and the JAK/STAT signaling pathway, are highlighted with red boxes. **E** Heatmap showing the expression of expert knowledge-selected STAT5 target genes, including JPX-1244 treated and DMSO control samples after 24 h without IL-6 stimulation. Unsupervised hierarchical clustering revealed a striking separation between treated and untreated T-PLL cases. Results were confirmed in an unbiased approach, utilizing the KEGG JAK/STAT gene list (Supplementary Fig. 4A). The patient identification codes (as summarized in Supplementary Table 2) are displayed in the legend at the top.

To investigate the downstream effects of JPX-1244-mediated inhibition of the transcription factors STAT3 and STAT5 in T-PLL, we conducted RNA sequencing of eleven T-PLL cases (7 HR and 4 LR samples) upon treatment with sublethal 2.4 µM JPX-1244. We sequenced 8 conditions for each case: an 8 h and 24 h timepoint, each dichotomized by DMSO vehicle control vs. JPX-1244 treatment, of which each was maintained unstimulated vs. stimulated with 2 ng/ml IL-6 for the above durations (Fig. 4A). Upon JPX-1244 treatment, 1 311 genes were significantly differentially expressed at 8 h and 4 801 genes at 24 h of treatment, compared to the respective untreated controls (FDR < 0.05), with an overlap of 1 220 genes between both timepoints (24.9% of all DEGs, Fig. 4B). Notably, we observed that 93.06% of genes that were differentially expressed after 8 h of JPX-1244 treatment, were also found differentially expressed after 24 h of treatment, while the vast majority (73.2%) of DEGs after 24 h of JPX-1244 treatment were only detectable after 24 h. Exemplarily, we determined upregulation of the TCR checkpoint inhibitor *CTLA4* and downregulation of the STAT5 target gene *FGL2* after 8 h treatment, and upregulation of genes encoding for heat shock proteins like *HSPB1* as signs of early apoptotic signaling after 24 h of treatment (Fig. 4C). Upon IL-6 stimulation, we observed upregulation of *STAT3*, *JAK3*, and the negative feedback regulator *SOCS3* after 24 h (Supplementary Fig. 4A), a pattern that was previously described for this signaling axis [43]. JPX-1244-mediated effects were comparable between the unstimulated and IL-6 stimulated conditions (Supplementary Fig. 4B, C). By conducting GSEA (Hallmark gene sets) of DEGs in unstimulated T-PLL cases upon JPX-1244 treatment, we identified significant enrichment of the PI3K-AKT-mTOR signaling pathway, the p53 pathway, apoptotic signaling, IL-2-STAT5, and IL-6-STAT3 gene sets at both 8 h and 24 h timepoints (FDR < 0.05, Fig. 4D).

Next, we evaluated the specificity of JPX-1244 treatment on STAT5 target genes. We performed unsupervised hierarchical clustering analyses of untreated and JPX-1244-treated (24 h) T-PLL, utilizing an expert-knowledge-based gene list of STAT5 target genes (Fig. 4E) and an unbiased list of KEGG JAK/STAT genes (Supplementary Fig. 4D). We identified distinct clustering of untreated and JPX-1244-treated cases in both conditions, confirming JAK/STAT-specific alterations in the transcriptome of T-PLL cells upon treatment with JPX-1244.

### Association of JPX-1244 response with increased *TOX*, *PAK6*, and *SPINT1* expression

**Fig. 5:**
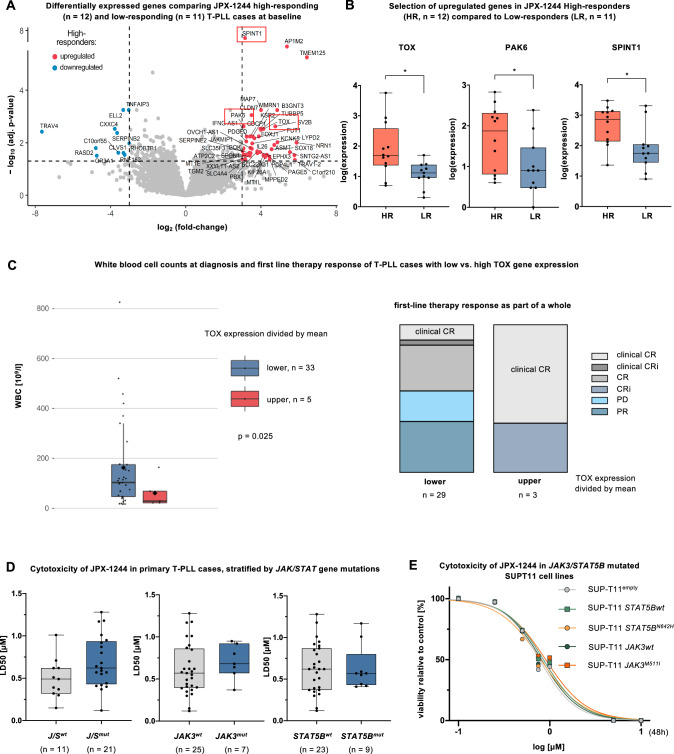
The sensitivity of T-PLL towards JPX-1244 is independent of *JAK/STAT* mutational status, but correlates with elevated *TOX, PAK6, and SPINT1* expression. **A** Volcano plot showing differentially expressed genes between JPX-1244 LR (*n* = 11) and HR (*n* = 12) T-PLL cases, as assessed by RNA-sequencing (without treatment or stimulation). Significantly upregulated genes in HR cases are marked in red and significantly downregulated genes in blue (cut-off at |log_2_FC|≧3 and FDR < 0.05). **B** Box plot showing *TOX*, *PAK6*, and *SPINT1* expression patterns, divided between HR and LR T-PLL cases (mean with minimum to maximum, two-tailed unpaired Student’s *t* test, *p < 0.05, FDR < 0.05). **C** Association of white blood cell counts (WBC) at diagnosis (left) and first-line therapy responses (right) with *TOX* mRNA expression, considering all T-PLL patients with data available on the respective clinical characteristic and *TOX* mRNA expression. T-PLL patients were divided in two groups based on the mean *TOX* mRNA expression. Left: WBC at diagnosis comparing low (*n* = 33, mean = 162.4 × 10^9^cells/l) and high (*n* = 5, mean = 61.1 × 10^9^cells/l) *TOX* expressing T-PLL cases (box plot with median as line, mean as rhombus, and whiskers as the interquartile range, *p* = 0.025, two-tailed unpaired Student’s *t* test). Right: Response to first-line therapy, divided in patients with low (*n* = 29) and high (*n* = 3) *TOX* expression (fisher’s exact test, *p* = 0.0121). Treatment responses were assessed as clinical complete remission (clinical CR), clinical complete remission with incomplete count recovery (clinical CRi), CR, CRi, progressive disease (PD), and partial response (PR), as previously defined [1]. **D** Association of JPX-1244 treatment responses with the *JAK/STAT* mutation status. A cohort of T-PLL patients (*n* = 32) was included, for whom cytotoxicity data from JPX-1244 treatment (see Fig. 2B) and corresponding *JAK/STAT* mutation profiles (see Fig. 1) were available. Box plots of JPX-1244 LD50 values, divided between T-PLL patients (left) with and without any mutation in a JAK/STAT family member (left: without ‘*J/S wt*’ *n* = 11, mean = 0.49 µM; with ‘*J/S mt*’ *n* = 21, mean = 0.69 µM), between T-PLL patients with and without JAK3 mutation (middle: without ‘*JAK3 wt*’ *n* = 25, mean = 0.60 µM; with ‘*JAK3 mt*’ *n* = 7, mean = 0.70 µM), and between T-PLL patients with and without STAT5B mutation (right: without ‘*STAT5B wt*’ *n* = 23, mean = 0.64 µM; with ‘*STAT5B mt*’ *n* = 9, mean = 0.62 µM). Mean with whiskers minimum to maximum is presented. No significant differences were observed in all three comparisons (*p* > 0.05, two-tailed unpaired Student’s *t* test). **E** Dose-viability curves of the TCL1A positive T-PLL-like cell line SUP-T11 upon transgenic *JAK/STAT* alterations. The strains SUP-T11^empty^, SUP-T11 STAT5B^wildtype^, SUP-T11 *STAT5B*^*N642H*^, SUP-T11 JAK3^wildtype^, SUP-T11 *JAK3*^*M511I*^, were treated with increasing concentrations (0.1 µM to 10 µM) of JPX-1244 and viability was assessed via AnnexinV/7AAD flow cytometry after 48 h.

As neither basal nor IL-6-induced pSTAT3 and pSTAT5 levels could be associated with JPX-1244 responses (Supplementary Fig. 5), we aimed to identify potential factors for lower responsiveness towards JPX-1244. For this purpose, we compared the transcriptomes of the T-PLL samples categorized based on their sensitivity to JPX-1244, namely 12 HR and 11 LR patients. In total, we identified 11 downregulated and 45 upregulated genes in HR cases compared to LR patients (FDR < 0.05, Fig. 5A). Among the significantly upregulated genes in the HR cohort, we identified *TOX* (*p* = 0.0110), a transcription factor described to regulate T-cell differentiation and to prevent T-cell overstimulation [44], *PAK6* (*p* = 0.0392), a transcriptional regulator promoting cell survival with overexpression in various tumors [45], and *SPINT1* (*p* = 0.0141, all Student’s *t* test), a protease inhibitor and negative prognostic marker for treatment response in chronic lymphocytic leukemia [46] (Fig. 5B). Further substantiating a potential implication of these gene deregulations in T-PLL, we performed correlations between the respective gene expression and clinical characteristics. Interestingly, T-PLL cases with higher *TOX* expression tended to exhibit lower white blood cell counts at diagnosis (T-PLL cases with lower *TOX* expression *n* = 33, vs. higher *TOX* expression *n* = 5, *p* = 0.025, Student’s *t* test, Fig. 5C) and displayed better responses to first-line therapies (T-PLL with lower *TOX* expression *n* = 29, vs. higher *TOX* expression *n* = 3, *p* = 0.0121, Fisher’s exact test). No significant associations between *PAK6* and *SPINT1* expression and clinical features were observed.

### JPX-1244 treatment responses are independent of *JAK/STAT* mutation status

Given the high prevalence of mutations in *JAK* or *STAT* genes in T-PLL, we sought to determine whether the *JAK/STAT* mutation status impacts JPX-1244 sensitivity. *JAK/STAT* mutation status, as assessed by RNA variant calling, was associated with 48 h ex-vivo JPX-1244 treatment responses in the cohort of 32 T-PLL. We detected no significant differences in LD50 values between T-PLL cases without a mutation in any *JAK/STAT* gene (*n* = 11, mean LD50 = 0.49 µM) vs. patients with any *JAK/STAT* mutation (*n* = 21, mean LD50 = 0.69 µM, Fig. 5D). We further investigated single mutations, but neither *JAK3* mutated (*n* = 7, mean LD50 = 0.70 µM) nor *STAT5B* mutated cases (*n* = 9, mean LD50 = 0.64 µM) displayed significantly different JPX-1244 LD50 as compared to cases with *JAK3* (*n* = 25, mean LD50 = 0.60 µM) or *STAT5B* (*n* = 23, mean LD50 = 0.62 µM) in wildtype configuration. As most *STAT5B* mutations cluster in the SH2 domain (Supplementary Fig. 1B), this finding remarkably indicates an independence of JPX-1244 treatment efficacy from SH2-phoshphopeptide affinity.

To further confirm the independence of JPX-1244 activity from the presence of *JAK/STAT* GOF mutations, we evaluated responses in the T-PLL-like cell line SUP-T11, that was modified by carrying either a *STAT5B*^*N642H*^ or a *JAK3*^*M511I*^ mutation. Following 48 h of treatment with JPX-1244, AnnexinV/7AAD flow cytometry revealed no significant differences in cell death induction between the *STAT5B*-mutated, *JAK3*-mutated, and unmutated variants (Fig. 5E). These findings align with our hypothesis that constitutive JAK/STAT activation is independent of specific *JAK/STAT* mutations, making STAT3/STAT5 inhibition a promising target for T-PLL cases, irrespective of their *JAK/STAT* molecular profile.

### Cladribine, venetoclax, and azacytidine as effective partners of specific STAT3/STAT5 inhibition

**Fig. 6:**
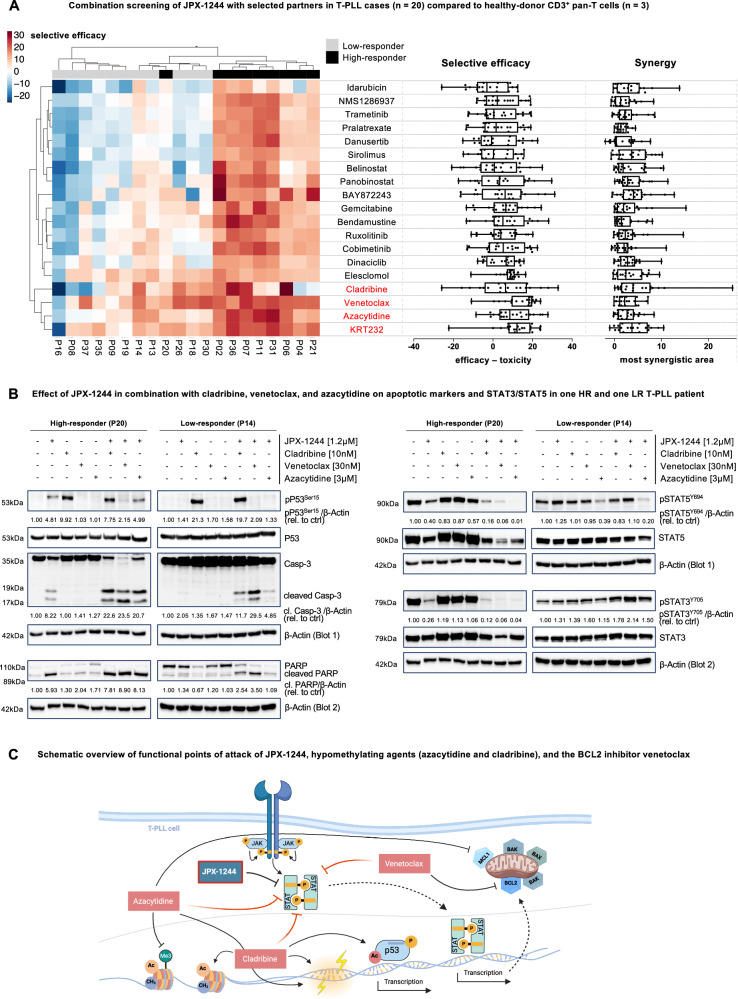
Combination screening identifies cladribine, venetoclax, and azacytidine as effective partners for JPX-1244 towards promising dual inhibitory concepts. **A** Combination screening in 20 T-PLL cases and 3 healthy controls (CD3^+^ T cells and PBMC). JPX-1244 was combined with 19 combination partners, comprising 8 compounds chosen based on (i) T-PLL’s pathogenesis and previous trials [30, 31, 47, 48]: KRT-232, belinostat, ruxolitinib, cladribine, bendamustine, trametinib, dinaciclib, and azacytidine, and (ii) a computational framework, predicting 11 compounds to transform the transcriptome of LR patients to resemble the one of HR patients [49]: elesclomol, danusertib, BAY872243, panobinostat, idarubicin, gemcitabine, sirolimus, pralatrexate, cobimetinib, venetoclax and NMS1286937. T-PLL samples and healthy controls were treated for 48 h, with 6 increasing concentrations of each compound (see Supplementary Table 4 for concentrations), a 1:1 combination was used in pairwise drug combination testing with JPX-1244, followed by predictions with the DECREASE machine learning model to fill the full (7 ×7) drug combination dose-response matrices [57]. Cell viability was assessed via CellTiter-Glo luminescent assay. Efficacy scores and synergy were calculated as previously described [31]. Left: Heatmap showing color-coded selective efficacy of 19 combination therapies, presented per patient (red: selective efficacy score >0, white: selective efficacy score =0, blue: selective efficacy score <0). Selective efficacy scores were calculated with the toxicity volume score and efficacy volume score using the SynToxProfiler [58]. The combinations were tested in 20 T-PLL patient samples and in CD3^+^ T cells derived from 3 age-matched healthy donors. For the selective efficacy in T-PLL samples compared to PBMC of healthy donors, see Supplementary Fig. 6B. Top legend displays the response status of each T-PLL patient towards JPX-1244 in previous single compound screenings (see Fig. 3C, gray: low-responder *n* = 11, black: high-responder *n* = 9). Unsupervised hierarchical clustering of both combination partners and T-PLL cases was performed. The respective patient ID is given in the bottom legend. The respective compound combined with JPX-1244 is given on the right. Middle: Selective efficacy values of 19 combination therapies in 20 T-PLL cases displayed as box plot (mean with minimum to maximum). Right: Box plot showing the most synergistic area of 19 combination therapies in 20 T-PLL cases (mean with minimum to maximum). For detailed information on the conditions of the combination screening and calculation of efficacy and synergy, see Supplementary Methods. **B** Immunoblots of one HR (P20) and one LR (P14) T-PLL case, upon 24 h treatment with JPX-1244 (1.2 µM), cladribine (10 nM), venetoclax (30 nM) and azacytidine (3 µM), or the combination of JPX-1244 with either cladribine, venetoclax or azacytidine, with the same sublethal dosages as in the mono-therapy condition, compared to DMSO control. Left: pP53^Ser15^ and P53, Caspase-3 (Casp-3) and cleaved (cl.) Caspase-3, PARP, and cleaved PARP were stained as markers for apoptotic signaling. Densitometric quantification of pP53^Ser15^, cleaved Caspase-3 and cleaved PARP was calculated normalized to the housekeeper β-Actin and relative to the DMSO control (rel. to ctrl). Right: pSTAT5^Y694^, STAT5, pSTAT3^Y705,^ and STAT3 signals are shown, quantified relative to the DMSO control, and normalized to the housekeeper β-Actin. As two different gels were used, the housekeeper β-Actin is shown below the respective proteins (Blot 1: pP53^Ser15^, P53, Caspase-3, pSTAT5^Y694^ and STAT5; Blot 2: PARP, pSTAT3^Y705,^ and STAT3). **C** Schematic overview showing functional points of attack of dual STAT3/STAT5 inhibition and the combination partners cladribine, venetoclax, and azacytidine. JPX-1244 directly targets STAT3 and STAT5, affecting both protein phosphorylation and stability. Azacytidine carries demethylating functions by inhibiting the DNA methyltransferase [59], induces DNA damage by incorporation in DNA and RNA [60], and inhibits MCL-1 and BCL-XL expression [61]. Cladribine induces DNA strand breaks as an analog of the nucleoside deoxyadenosine [62], is able to evoke P53 activation [31], and harbors hypomethylating functions as well [63]. Venetoclax selectively binds and antagonizes BCL2, by mimicking the BH3 domain of pro-apoptotic proteins [64]. The effects on these known targets are shown as black, solid arrows, and the interrupted physiological signaling cascades as black, dotted arrows. We propose a synergism of these three compounds with JPX-1244 through indirect effects, exemplarily through hypomethylation of regulators or effects on BCL2 family members, or through direct effects on STAT3/STAT5 phosphorylation (red inhibitors), ultimately leading to decreased expression of STAT target genes. Graph was created with Biorender.

Enhancing the efficacy of single-compound therapies often comes at the expense of increased toxicity, highlighting the need for well-selected combination approaches. We, therefore, implemented a two-pronged strategy to identify optimal partners for JPX-1244: (i) compound selection based on the current understanding of T-PLL pathogenesis [31, 47] and prior ex-vivo drug testing [30, 48] identifying KRT-232, belinostat, ruxolitinib, cladribine, bendamustine, trametinib, dinaciclib, and azacytidine. In addition, (ii) we utilized RNA sequencing data to predict compounds capable of reprogramming the transcriptome of LR patients to resemble that of HR patients, leveraging a novel machine learning framework [49]; this identified elesclomol, danusertib, BAY872243, panobinostat, idarubicin, gemcitabine, sirolimus, pralatrexate, cobimetinib, venetoclax, and NMS1286937. In the subsequent ex-vivo screening, we investigated the efficacy and selectivity of these single compounds and their combination with JPX-1244 in 20 T-PLL and in 3 healthy-donor derived controls (CD3^+^ T cells and PBMC) after 48 h of treatment (see Supplementary Table 4 for concentrations). Elesclomol and KRT-232 showed the highest drug sensitivity score (DSS) as single compounds (Supplementary Fig. 6A). In combination with JPX-1244, the DNA-methyl transferase inhibitor azacytidine, the hypomethylating nucleoside cladribine, the BCL-2 inhibitor venetoclax, and the MDM-2 inhibitor KRT-232 emerged as top candidates (Fig. 6A). Combinations with these compounds not only demonstrated high potency against T-PLL, but also, through unsupervised hierarchical clustering, tend to ameliorate the previous JPX-1244-defined differences between HR and LR patients. Importantly, these 4 combinations also exhibited strong T-PLL selectivity as compared to T cells (Fig. 6A) and PBMCs from healthy donors (Supplementary Fig. 6B).

In a pilot experiment, we then investigated synergistic mechanisms underlying the efficacy of three selected combinations. We treated primary T-PLL cells derived from one HR and one LR patient with sublethal dosages of JPX-1244 (1.2 µM), cladribine (10 nM), venetoclax (30 nM) and azacytidine (3 µM) for 24 h, as mono-therapies or in combination. Apoptotic markers, including cleavage of PARP and Caspase-3, were prominently induced in the HR patient and also became evident in the LR patient when treated with the combinations (Fig. 6B). Furthermore, we observed pronounced phosphorylation of P53 upon treatment with cladribine in both the HR and the LR patient. Interestingly, phosphorylation of P53 was also prominent upon treatment with JPX-1244 as a single-compound as well as in combination with azacytidine in the HR patient. Next, we investigated the effect of cladribine, azacytidine, and venetoclax on the JPX-1244 targets STAT3 and STAT5. Remarkably, azacytidine as a single agent reduced STAT5 phosphorylation in both the HR and the LR patient, and this effect was significantly amplified when combined with JPX-1244. In line, we observed a similar effect on STAT5 and STAT3 phosphorylation by the combination of JPX-1244 with cladribine or venetoclax.

These findings establish through predictions and validations the agents cladribine, azacytidine, and venetoclax as synergistic and T-PLL-specific combination partners for JPX-1244.

## Discussion

Our analysis highlights constitutively active JAK/STAT signaling as a central pathogenetic lesion and an actionable target in T-PLL. Based on this initial rationale, we evaluated novel dual STAT3/STAT5 non-PROTAC degraders in T-PLL. The marked anti-T-PLL cell potency and selectivity of the lead compound JPX-1244 was paralleled by its specific effects on STAT3/STAT5 phosphorylation, protein stability, and STAT5 target gene expression. Combining the degrader with agents such as cladribine, azacytidine, and venetoclax enhanced efficacy through synergistic effects on STAT5 phosphorylation, even in samples with lower single-agent sensitivities, adding to the translational potential of STAT3/STAT5 inhibition as a novel strategy in T-PLL.

Augmenting the findings of our 2019 meta-analysis [20] by including 60 newly characterized T-PLL towards a cohort of 335 cases, we detected gene mutations in at least one JAK or STAT family member in 52.4% of T-PLL, most commonly in *JAK3*, *STAT5B*, *STAT2*, and *JAK1*, representing a slight decrease from the prior 62.1% [20], possibly attributable to lower sensitivity of RNA-sequencing [50, 51]. We also report recurrent aberrations in the expression profile of JAK/STAT regulators.

Interestingly, we identified *STAT2* mutations in 8.5% of cases. Such lesions have not yet been described in T-PLL. *STAT2*, typically associated with antiviral immunity and inflammatory regulation, has also been implicated in carcinogenesis, possibly through IL-6-mediated STAT3 activation [52, 53]. The location of the most recurrent *STAT2* mutations in our cohort in the C-terminal transactivation domain might indicate a loss of function, which is interesting in the tumor-suppressing context described for STAT2 [54]. Future studies, e.g. those on target genes of *STAT2*-mediated transcription in T-PLL, are needed.

We demonstrate promising therapeutic potential of dual STAT3/STAT5 non-PROTAC degraders from the JPX-series, which effectively inhibited STAT3/STAT5 phosphorylation and induced protein degradation, both underlying a marked cell death response. Notably, transcriptomic responses to such STAT3/STAT5 inhibition were accompanied by specific changes in STAT5 target gene levels, similar to those seen in AML and natural killer/T cell lymphoma (NKCL) [35].

Attempts to target JAK/STAT signaling in T-PLL are not new and there are sporadic clinical responses reported for JAK inhibitors or their combination with venetoclax in relapsed T-PLL [26–29]. However, inhibiting the upstream JAK molecules did not exhibit encouraging anti-leukemic efficacy in larger compound screenings [30, 31]. The superior therapeutic efficacy of dual STAT3/STAT5 degraders observed here likely originates from (i) an elevated (although somewhat variable) dependence of primary T-PLL cells on STAT signaling, (ii) the position of STATs distal of JAKs, allowing for addressing modes of activation by JAK-independent mechanisms (e.g. GOF *STAT* mutations), and (iii) the suppression of bypass mechanisms such as SOCS2-mediated STAT3 activation upon STAT5 inhibition [32, 33]. This argues for future systematic studies of STAT3/STAT5 targeting instead of JAK inhibition in T-PLL, particularly since our data also suggest that JPX-1244 sensitivities are independent of *JAK/STAT* mutations.

Recognizing the limitations of monotherapies, such as toxicity and resistance, we conducted a combination screening using JPX-1244 alongside 19 potential combination partners. To avoid bias from relying solely on ‘expert knowledge’, we complemented our selection with a machine learning framework that predicted compounds capable of in-silico converting the gene expression profiles of low-response (LR) patients toward those of high-response (HR) patients [49]. Notably, the hypomethylating agents azacytidine and also cladribine, the BCL-2 inhibitor venetoclax, and the MDM2 inhibitor KRT-232 emerged as top candidates. These agents not only demonstrated substantial efficacy, but also tended to resolve the clustering of JPX-1244 LR and HR patients. Combinations of JPX-1244 with the hypomethylating agents or with venetoclax further effectively reduced STAT3 and STAT5 phosphorylation. A synergistic relationship of STAT3-targeting and cladribine has so far only been described in multiple myeloma [55]. The mechanisms underlying the effects on STAT3/STAT5 phosphorylation could be indirect, e.g. (i) transmitted through the hypomethylating functions of azacytidine and cladribine, e.g. on regulators such as SOCS protein family members, or (ii) through the suppressive role of azacytidine and venetoclax on BCL2 family members, or they could be a direct impact on STAT phosphorylation (Fig. 6C). Furthermore, it has previously been shown that *STAT3* GOF mutations cause DNA hypermethylation, resulting in high sensitivity towards azacytidine in large granular lymphocyte leukemia (LGLL) [56], which could contribute to the potency of hypomethylating agents in a disease characterized by JAK/STAT activation such as T-PLL. These potential models of synergism require further functional validation, but the findings highlight promising therapeutic avenues. Future studies should additionally validate the efficacy of these combinations in vivo, for instance, using patient-derived xenograft (PDX) models of T-PLL.

In conclusion, we present dual STAT3/STAT5 inhibition as a promising, novel therapeutic strategy for T-PLL, demonstrating potent and T-PLL-selective anti-leukemic efficacy. Particularly combinations with hypomethylating or BCL2-targeting agents have the potential to address the unmet therapeutic needs in this problematic disease.

## Supplementary information

Supplementary Material

Supplementary Table 1

Supplementary Table 2

Supplementary Table 3

Supplementary Table 4

## Data Availability

The bulk RNA sequencing data newly generated within this study have been deposited in the Gene Expression Omnibus under accession code GSE286270 (reviewer accession code: gnopwkoonxypnyt).
