# Supplementary material for: Dual STAT3/STAT5 inhibition as a novel treatment strategy in T-prolymphocytic leukemia
Source: Leukemia. 2025 Apr 15;39(6):1435–48. doi: 10.1038/s41375-025-02577-8 (PMC12133589; doi:10.1038/s41375-025-02577-8)
Supplement: Supplementary file 1 — Supplementary Material [file 41375_2025_2577_MOESM1_ESM.pdf]

Supplementary Materials: Dual STAT3/STAT5 inhibition as a novel treatment strategy in T-prolymphocytic leukemia

<u>**Inventory**</u>

- Supplementary Materials and Methods
- Supplementary Figures 1-6
- Supplementary References

Supplementary information in separate files:

- Supplementary Table 1: Distribution of T-PLL cases and sequencing methods included in the meta-analysis on *JAK/STAT* mutations
- Supplementary Table 2: Patient characteristics of T-PLL cases included in *ex-vivo* experiments
- Supplementary Table 3: RNA expression of genes encoding for proteins potentially regulating JAK/STAT signaling in T-PLL cases (n=32) compared to $CD3^+$ T cells derived from healthy donors (n=6)
- Supplementary Table 4: Concentrations of compounds included in the combination screening of JPX-1244 with selected partners in T-PLL cases (n=20) and healthy-donor derived controls (n=3)

Supplementary Materials and Methods

Meta-Analysis

In this study, we included 275 T-PLL cases previously published in our meta-analysis in 2019(1), combined with additional 60 T-PLL cases, on which we performed RNA-sequencing to identify pathogenetic *JAK* or *STAT* variants. The additional 60 T-PLL cases consist of 30 re-analyzed T-PLL cases sequenced in 2020 and published in 2022(2), and 30 newly sequenced T-PLL cases. To exclude redundantly analyzed patients, we compared basic patient information and excluded 14 overlapping patients from originally 74 newly sequenced or re-analyzed T-PLL cases. Out of the 74 patients, 36 T-PLL cases (n=32 newly sequenced; n=6 re-analyzed) were utilized for *in-vitro* assessments of dual STAT3/STAT5 inhibition and transcriptomic analyses. To compare, we re-analyzed RNA sequencing data of $CD3^+$ T cells derived from 6 healthy donors, sequenced in 2020.

Together with our previously published cases, this resulted in 335 T-PLL patients with sequencing data on at least one *JAK* or *STAT* gene locus. In 147 patients, sequencing data on all JAK or STAT members were available. In the analysis of individual mutations, we included 306 cases with sequencing data on the *JAK1*, 332 cases on *JAK3*, 142 cases on *STAT2*, and 269 cases on the *STAT5B*. The publications, number of T-PLL cases derived from each and the respective sequencing method can be found in **Supplementary Table 1**. For details on the analysis of the previously published 275 T-PLL cases, please see the methodical description in our meta-analyses from 2019(1).

Patient cohort

In the *in-vitro* experiments, we included peripheral blood-derived samples of 58 T-PLL patients and 16 age-matched (>55 years) healthy donors. The diagnosis of T-PLL was confirmed according to WHO criteria and consensus guidelines(3). All patients provided informed consent according to the Declaration of Helsinki. The collection and use of samples were approved for research purposes by the Ethics Committee of the University Hospital of Cologne (#12-146, #19-089) or the University of Helsinki (303/13/03/01/2011). For detailed patient characteristics see **Supplementary Table 2**.

Cell isolation

Peripheral blood mononuclear cells (PBMCs) were isolated by density gradient centrifugation (#25-072-CV, Corning). $CD3^+$ T cells of age-matched healthy donors were derived from PBMCs by magnetic cell separation via negative selection (#480021, BioLegend) on LD Depletion Columns (#130-045-201, Miltenyi Biotec). Flow cytometry on CD3 (#300302, BioLegend) was performed to confirm purity. Samples with purity <90% were excluded.

Cell Culture

Primary isolates from T-PLL patients, healthy-donor-derived PBMC, as well as the T-cell lines Jurkat, HH, HuT78, and SUP-T11, were held in suspension cultures in Gibco™ RPMI-1640 medium supplemented by GlutaMAX™ (ThermoFisher, Waltham, USA), fetal bovine serum (FBS, #F0926500ML, Sigma-Aldrich; 10% for primary T-PLL cells, healthy-donor PBMC, and the T-cell lines Jurkat, HH, and HuT78; 20% for SUP-T11), and 1% Penicillin-Streptomycin (100U/1mM; #15140-111, Gibco). Alpha-MEM medium (#A-10490-0, Gibco) was used for culturing NKtert and KuSa bone-marrow stromal feeder cells. Suspension cells were maintained at a density of $2.0\text{-}8.0 \times 10^5$ cells/ml (cell lines) or 1.0×10^6 cells/ml (primary T-PLL cells, healthy-donor PBMC, and T cells). Adhesion cells (NKtert and KuSa) were maintained at a density of 5×10^5 cells/ml. For cocultures, the adhesive cells were plated at a density of 0.3×10^5 cells/ml, and proliferation was inhibited with Mitomycin C after 24h. Primary cells were added after an additional 24h at a density of 1.0×10^6 cells/ml. Cell cultures were kept in an incubator at 37°C, 5% CO_2, and 90% humidity. All cell lines were authenticated and regularly tested for Mycoplasma infection by standard PCR protocols. Cell viability and cell density were assessed via Trypan blue exclusion method using a Countess™ II automated cell counter (ThermoFisher).

In cytokine stimulations, cells were cultured with IL-2 (5ng/ml), IL-6 (2ng/ml), IL-7 (5ng/ml), IL-8 (1ng/ml), IL-15 (5ng/ml), TNF-a (2ng/ml) or medium alone at a density of 10^6 cells/ml for indicated timepoints. Stimulated cells were harvested on ice.

Single compound drug testing

The single-drug screenings included 28 JPX compounds and three compounds known in T-PLL treatment (bendamustine, ruxolitinib, and cytarabine). All compounds were diluted in DMSO (#4720.4, Carl Roth). T-cell lines and T-PLL cells were exposed to the compounds at indicated concentrations and timepoints, plated in 24 well plates, 48 well plates, or 10ml flasks at a density of 10^6 cells/ml. DMSO was added to the vehicle control condition. In the single compound screening of 28 JPX compounds, bendamustine, ruxolitinib and cytarabine in **Fig. 2A**, cell viability was assessed via CellTiter-Glo (CTG) luminescent assay (Promega). In viability assays in **Fig. 1F**, **Fig. 2B-2D**, **Supplementary Fig. 2D**, **Fig. 3B**, **Supplementary Fig. 3C**, and **Fig. 5D-E**, cell death was determined via AnnexinV-APC/7AAD flow cytometry (#640941, #420404, both BioLegend) according to standard protocols, expression was assessed on a Gallios cytometer using the Kaluza software (BeckmanCoulter).

Combination screening

In the combination screening, primary T-PLL cells were treated with JPX-1244 and 19 combination partners. We selected 8 compounds based on the current understanding of T-PLL pathogenesis(4,5) and prior ex-vivo drug testings(6,7): KRT-232, belinostat, ruxolitinib, cladribine, bendamustine, trametinib, dinaciclib, and azacytidine, and 11 based on a computational framework(8): elesclomol, danusertib, BAY872243, panobinostat, idarubicin, gemcitabine, sirolimus, pralatrexate, cobimetinib, venetoclax, and NMS1286937. T-PLL samples (n=20) and healthy controls ($CD3^+$ T cells and PBMC, n=3) were treated for 48h. For each drug combination, a diagonal dose-response design was tested experimentally using 6 increasing concentrations of each compound in 1:1 ratio of JPX-1244 with each partner drug. The full 7×7 dose-response matrices were then predicted using the DECREASE machine learning model (9). Cell viability was assessed via CellTiter-Glo luminescent assay. Compound sensitivity testing was performed as previously described(4,10). To quantify efficacy and selectivity, a drug sensitivity score (DSS) and a selective DSS (sDSS) were calculated as previously described(11,12). Combination synergy was scored with the ZIP synergy model using the SynergyFinder web-tool(13). Most synergistic area (MSA) corresponds to the 3 x 3 dose-response matrix with the highest synergy. The selective efficacy was calculated by subtracting the toxicity volume score from the efficacy volume score for every patient sample, using the SynToxProfiler web tool(14).

Experimental setup for western blots and RNA sequencing

Cell cultures of 11 T-PLL cases were performed for 8h or 24h. Each condition contained $1.5x10^7$ cells at a density of $1x10^6$/ml ($1x10^7$ cells for western blots, $5x10^6$ cells for RNA sequencing). Treatment with JPX-1244 was performed with a concentration of 2.4µM or DMSO control. Half of the samples were stimulated with 2ng/ml IL-6 (diluted in PBS, #200-06, Peprotech) for the indicated time period. After the incubation period, cells were carefully harvested on ice.

Immunoblotting

Western Blots on whole-cell protein lysates were performed according to standard protocols. Lysates were prepared on ice with PhosphoStop (1:50) and CompleteC (1:50) freshly added to NP40-based Lysis Buffer to preserve phosphorylation signals. Blots ran on 6%-15% gradient gels, were transferred via Semi-dry transfer blot (BioRad) and blocked with 4% BSA. The primary antibodies used were $pSTAT5^{Tyr694}$ (#9359 C11C5, Cell Signaling Technology, Danvers, USA), $pSTAT3^{Tyr705}$ (#9145 D3A7, CST), STAT5 (#25656S D3N2B, CST), STAT3 (#9139 124H6, CST), β-Actin (sc-1616, Santa Cruz Biotechnology, Dallas, Texas), phospho-$P53^{Ser15}$ (#9284, CST), P53 (#sc-126 DO-1, Santa Cruz Biotechnology), Caspase-3 (#9665 8G10, CST), and PARP (#9542L, CST), diluted 1:1000 in PBS-T, with 2% BSA, and 0.4% NaN_3. As peroxidase-labeled secondary antibodies, anti-mouse (#715-035-150, Dianova) and anti-rabbit (#711-035-152, Dianova) were used. Chemiluminescence was detected using Autoradiography Film Blue (Santa Cruz Biotechnology) and the developer machine CAWOMAT 2000 IR (CAWO Solutions, Schrobenhausen, Germany) or the Intas science imaging system (Intas ECL Chemostar). Densitometry was performed with the ImageJ software.

RNA isolation, library preparation, and sequencing

In total, 118 samples were sequenced. This includes RNA sequencing of untouched primary T-PLL samples (n=32, as described in the Meta-Analysis section), as well as eleven T-PLL patients (out of these 32 patients) in the context of JPX-1244 treatment (compared to untreated control) and/or IL-6 stimulation (compared to unstimulated control), resulting in 4 conditions per patient and timepoint. As we performed the treatment for 8 and 24h, this resulted in 86 samples in total (2 samples had to be excluded due to low RNA quality after treatment with JPX-1244). RNA was isolated using the Qiagen RNeasy Plus Mini Kit (#74134) according to the manufacturer's instructions. RNA quality and concentration were assessed using the 4150 TapeStation (Agilent, Santa Clara, USA).

RNA samples with RIN values below 5 were excluded. Library preparation was conducted with the Illumina TruSeq Stranded Total RNA Library Prep Gold Kit (rRNA-depleted total mRNA, #20020599). The samples were processed without ERCC RNA Spike-In controls. Paired-end sequencing (2x100bp) was performed with 50 million reads per sample.

RNA sequencing - data processing, analysis, and availability

RNA sequencing reads were aligned to the hg38 reference genome using Bowtie2 (version 2.3.5.1) with default settings(15). Quality control was performed using FastQC and MultiQC. Samtools 1.10 was utilized post-alignment for sorting and indexing the aligned reads(16). Gene-level counts were quantified using the Rsubread package (version 2.14.2) with the featureCounts function. Transcript variants were identified as previously described, using the GATK Best Practice workflow for RNA-seq variant calling (17). Variant annotation was performed using ANNOVAR (version 2020-06-08) (18). Maftools R package (v.2.16.0) was used to summarize variant annotations (19). Differential gene expression analysis was conducted using DESeq2 v1.34.0 with BH multiple testing correction (padj < 0.05) (20). Pathway enrichment analysis was performed using clusterProfiler (v.4.8.3) on differentially expressed genes using MSigDB (v2022.1.Hs) and KEGG gene sets (21). To identify potential therapeutic compounds, differentially expressed genes (FDR < 0.05) between low-response (LR) and high-response (HR) patients were provided to ScTherapy machine learning framework (8), to predict drugs capable of reversing the LR expression profile toward the HR profile.

SUP-T11 cell lines

The SUP-T11 cell line was transfected to express FLAG-tagged human STAT5B^{wildtype}, STAT5B^{N642H}, JAK3^{wildtype}, or JAK3^{M511I}, using an amphotropic retrovirus vector system and relying on standard operating conditions. HEK293-derived HekGP cells, stably expressing the Gag-Pol proteins, were transfected using Lipofectamine 2000 (Thermo Fisher Scientific) with the pMD2.G plasmid (Addgene, #12259) for the vesicular stomatitis virus glycoprotein (VSV-G) envelope, and the pMSCV-IRES-GFP transgene constructs. SUP-T11 cells were transduced with the first harvest of viral supernatants, in the presence of polybrene (8µl/ml) to enhance infection efficacy, by spinfection at 900 x g for 45min at room temperature. Following transduction, cells were cultured in Gibco™ RPMI-1640 medium, supplemented with 20% fetal calf serum (FCS), 2mM L-glutamine (Gibco™, #25030123), 1% penicillin-streptomycin, and Normocin (InvivoGen). At Day 5 post-transduction, GFP-positive cells were sorted using the BD

FACS ARIA III cell sorter. Cells were maintained in RPMI medium supplemented as described above, with Normocin included for an additional two weeks. Sorting was performed three times. Cells were cultured in six-well plates between sorts and monitored for viability and expression stability.

Statistics

Numbers of biological replicates, p-values and information on means and statistical tests are included in the respective figure, where needed. Due to the exploratory nature of the study, no formal sample size calculation was performed. If not marked otherwise, the standard deviation (SD) as error bars and the mean are given. When comparing one variable of two distinct groups, a two-tailed unpaired Student's t-test was used as a statistical test. An F-test was performed to assess variance homogeneity, and if significant, a Welch correction was applied. When comparing one variable of one group at different conditions, a two-tailed paired Student's t-test was used. When comparing multiple variables of distinct groups, a two-way ANOVA with Geisser-Greenhouse correction and Bonferroni's multiple comparisons test was used. Normal distribution was assessed using the Kolmogorov-Smirnov test, and if the data were not normally distributed, a Wilcoxon test was performed instead of a tow-tailed paired Student's t-test. P-values <0.05 (*) were considered significant.

Data availability

The bulk RNA sequencing data newly generated within this study have been deposited in the Gene Expression Omnibus under accession code GSE286270 (reviewer accession code: gnopwkoonxypnyt).

Supplementary Figures

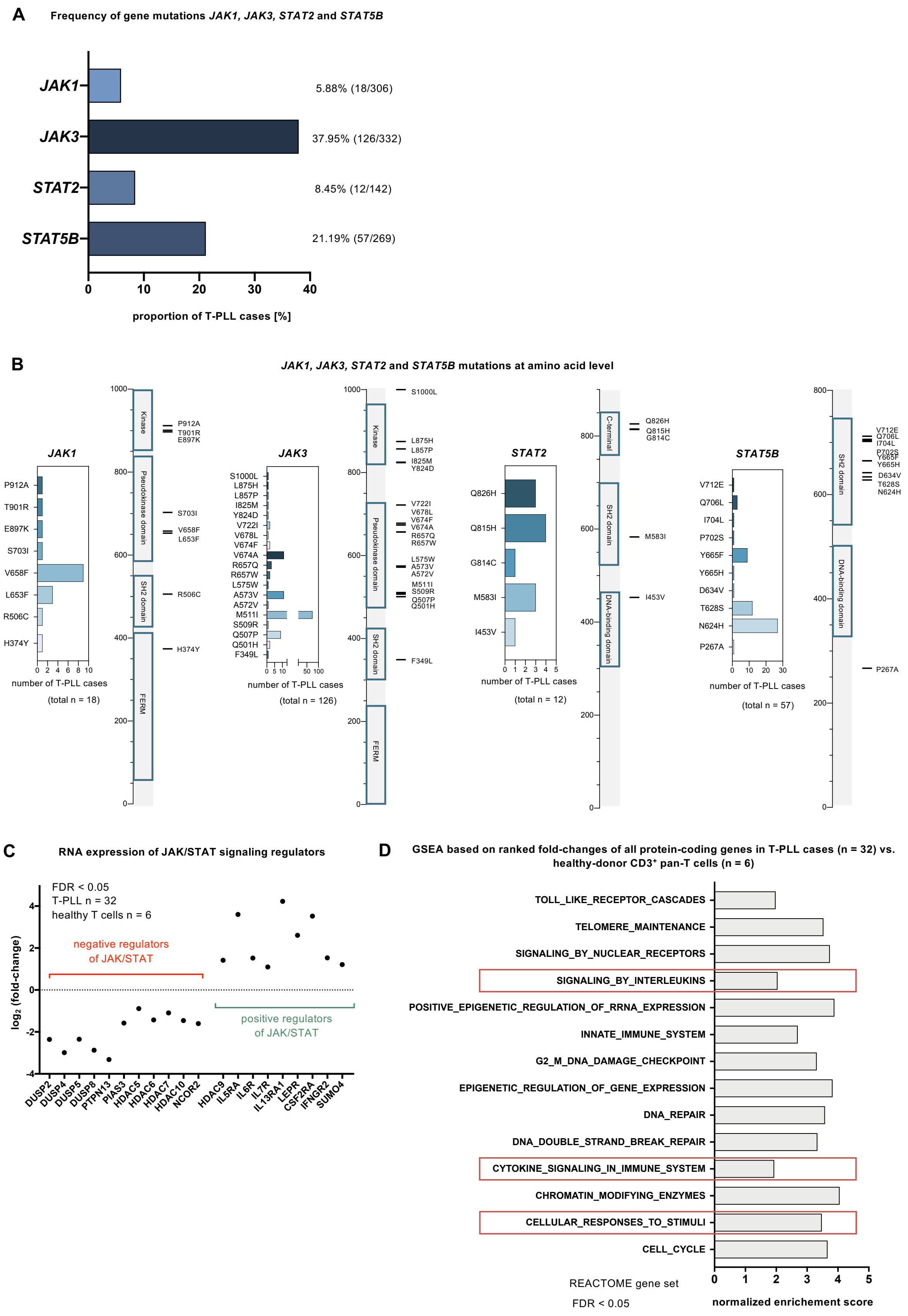

Supplementary Figure 1: Most prevalent *JAK/STAT* mutations, RNA expression of JAK/STAT regulators and enriched cytokine signaling pathways in primary T-PLL cells

A We expanded a previously published meta-analysis containing sequencing data on 275 T-PLL cases(1) with 60 additional cases, assessed for JAK/STAT mutations by RNA profiling, resulting in a cohort of 335 T-PLL patients with sequencing data on at least one *JAK* or *STAT* gene locus. Frequency of mutations in *JAK1, JAK3, STAT2*, and *STAT5B*. All patients with available sequencing data on the respective gene were included (*JAK1* n=306 T-PLL patients, *JAK3* n=332, *STAT2* n=142, *STAT5B* n=269). **B** Location and frequency of mutations in *JAK1*, *JAK3*, *STAT2,* and *STAT5B* on amino acid level. Left: Horizontal bar chart showing the frequency of the respective mutation in absolute numbers of T-PLL cases. All T-PLL cases with sequencing data on the respective mutated gene are included (total number of mutated cases included: *JAK1* n=18, *JAK3* n=126, *STAT2* n=12, *STAT5B* n=57). Right: Simplified schematic view of the molecular structure of each protein and its domains, distributed along the amino acid numbers, with each detected mutation placed at its location. **C** RNA expression levels of JAK/STAT regulators in T-PLL cases (n=32) compared to $CD3^+$ T cells derived from age-matched healthy donors (n=6). JAK/STAT regulators have been selected expert knowledge-based and are listed in **Supplementary Table 3**. Only genes differentially expressed with an FDR<0.05 were included in the graph. **D** Gene set enrichment analysis (GSEA) derived from RNA sequencing of 36 T-PLL cases, compared to $CD3^+$ T cells from 6 healthy donors. The REACTOME gene sets were used, and only gene sets significantly enriched with an FDR<0.05 were included in the graph. Gene sets particularly interesting in the context of cytokine signaling are highlighted in red boxes.

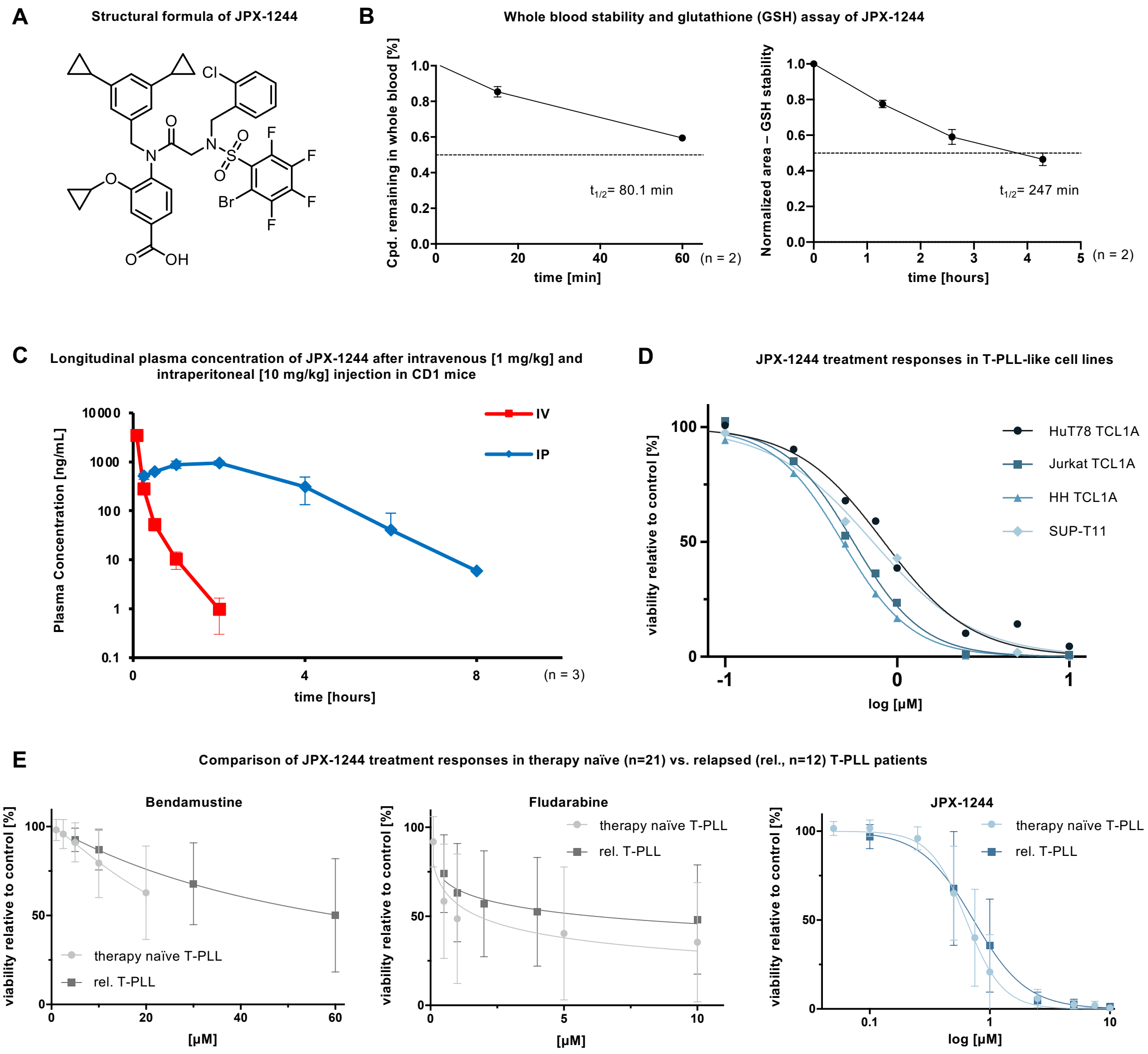

Supplementary Figure 2: JPX-1244 exhibits strong *ex-vivo* and *in-vivo* stability and induces cell death in T-PLL-like cell lines and relapsed T-PLL patients

A Structural formula of Non-PROTAC STAT3/STAT5 degrader JPX-1244. **B** Stability and safety data on JPX-1244 *in vitro*. Left: Whole blood stability of JPX-1244 in human blood, half-life was 80.1min (n=2, mean with SD). Right: Glutathione (GSH) stability of JPX-1244 in IMDM + 10% FBS, half-life of GSH was 247min (n=2, mean with SD). **C** *In vivo* plasma concentration of JPX-1244 in CD1 mice (n=3) upon intravenous (IV, 1mg/kg) or intraperitoneal (IP, 10mg/kg) injection. Mean half-life of JPX-1244 upon IV injection was 16min, mean half-life of JPX-1244 upon IP injection was 37min (mean with SD given in the graph). **D** Dose-viability curves of JPX-1244 in 4 T-PLL-like cell lines HuT78 TCL1A, Jurkat TCL1A, HH TCL1A, and SUP-T11. Viability was assessed after 48h of treatment with increasing concentrations of JPX-1244 via AnnexinV/7AAD flow cytometry. **E** Dose-viability curves of bendamustine, fludarabine, and JPX-1244 after treatment of 21 therapy naïve T-PLL cases (light grey or light blue) compared to 12

relapsed (rel.) T-PLL patients (dark grey, dark blue) for 48h with increasing concentrations of each compound. Viability was assessed via AnnexinV/7AAD flow cytometry. Mean with SD is shown.

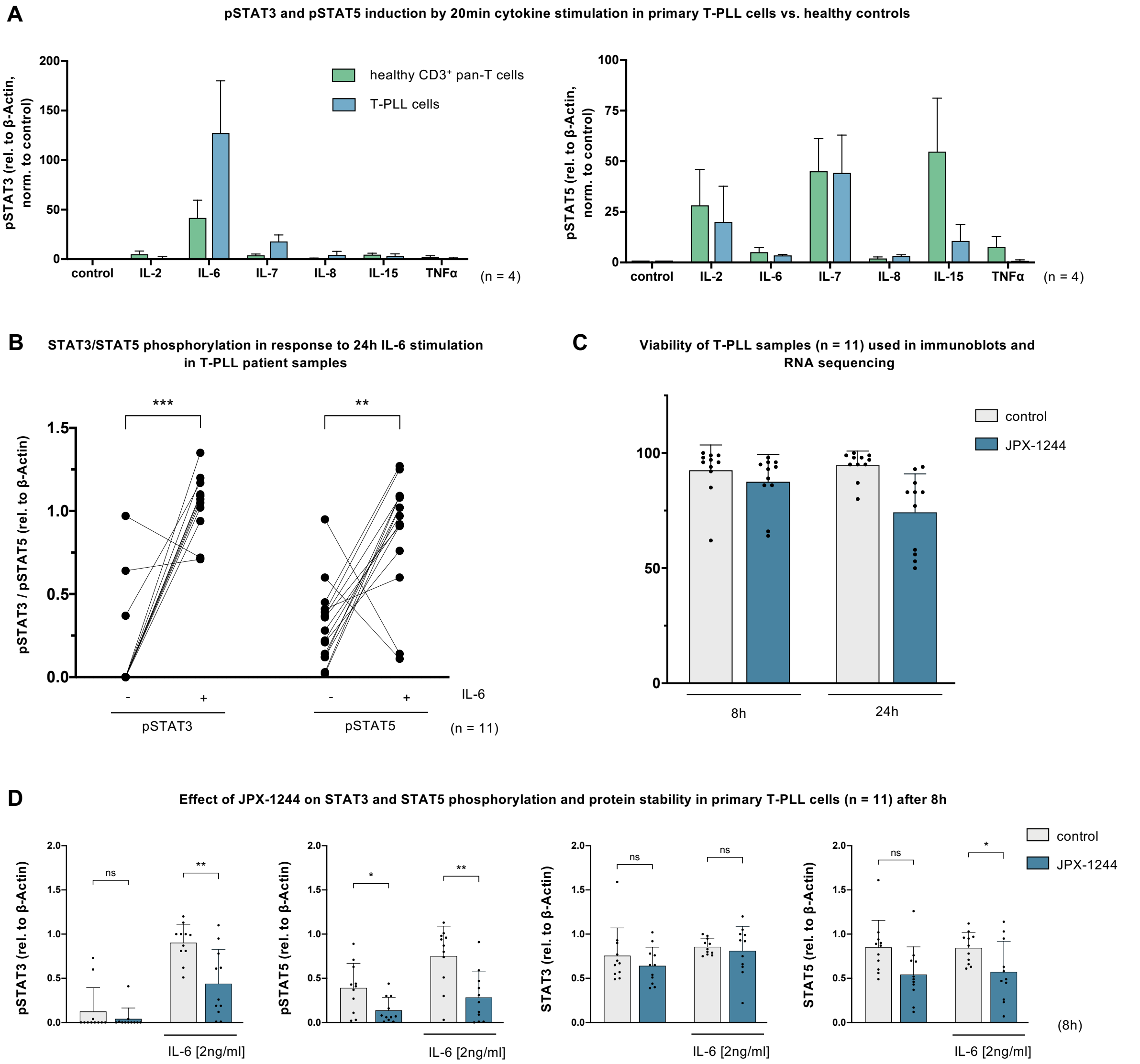

Supplementary Figure 3: STAT3 and STAT5 phosphorylation upon cytokine stimulation as well as JPX-1244 treatment in primary T-PLL cells.

A Densitometry of pSTAT3 and pSTAT5 in 4 T-PLL cases and CD3⁺ T cells derived from 4 healthy donors, upon 20min cytokine stimulation with IL-2, IL-6, IL-7, IL-8, IL-15 and TNFα. Detailed information on the concentrations and immunoblots are given in the **Supplementary Methods**. Left: Quantification of pSTAT3 signals in immunoblots of T-PLL cases compared to healthy CD3⁺ T cells, normalized to the housekeeper protein β-Actin and to the control condition (mean with SEM). Right: Quantification of pSTAT5 signals in immunoblots of T-PLL cases compared to healthy CD3⁺ T cells, normalized to β-Actin and to the control condition (mean with SEM). **B** Densitometry of pSTAT3 and pSTAT5 signals after 24h, unstimulated or stimulated with 2ng/ml IL-6. All 11 T-PLL cases included in the immunoblots in **Fig. 3A, C-D** are shown, and the respective points of each individual patient are linked (aligned dot plot, tow-tailed paired Student's t-test, **p<0.01, ***p<0.001). **C** Viability of the 11 T-PLL cases used in

the immunoblots in **Fig.3A, C-D** and the RNA-sequencing in **Fig.4**, after 8h or 24h treatment with 2.4µM JPX-1244, compared to the DMSO control. Viability was measured via trypan blue exclusion (mean with SD). **D** Densitometry of pSTAT3, pSTAT5, STAT3 and STAT5 signals in 11 T-PLL patients, untreated or upon treatment with 2.4µM JPX-1244 for 8h, unstimulated or stimulated with 2ng/ml IL-6, assessed by the quantification in immunoblots and normalized to β-Actin (mean with SD, two-tailed paired Student's t-test, non-significant (ns), *$p<0.05$, **$p<0.01$).

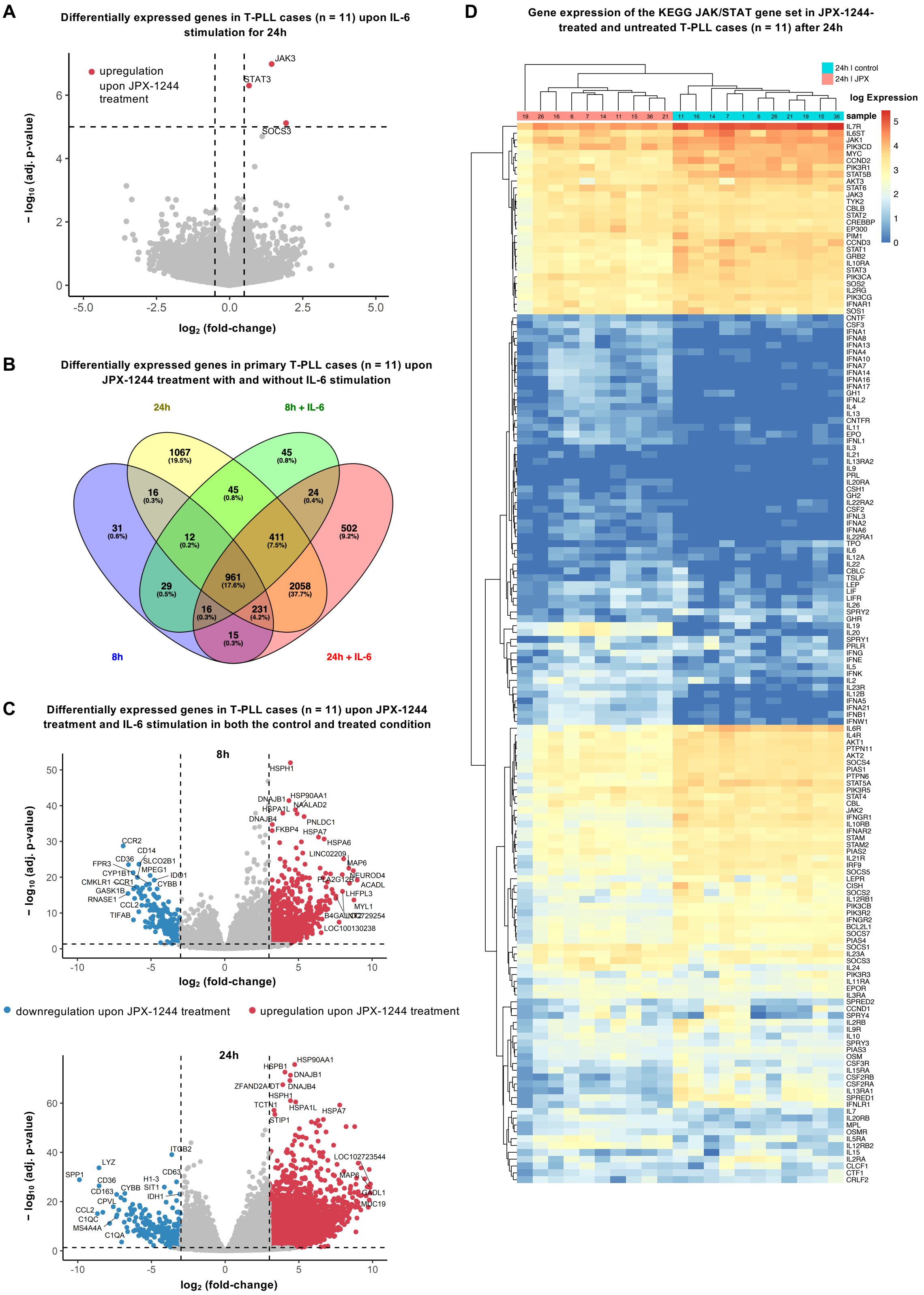

Supplementary Figure 4: Transcriptomic alterations in primary T-PLL cells upon JPX-1244 treatment and IL-6 stimulation

A Volcano plot showing differentially expressed genes (DEG) in 11 T-PLL cases upon stimulation with 2ng/ml IL-6 for 24h, compared to the unstimulated control after 24h. Significantly upregulated genes are

marked as red dots ($|log_2FC| \geqq 0.6$ and an FDR<$0.08x10^{-6}$). **B** Venn diagram displaying the overlap of differentially expressed genes upon 2.4µM JPX-1244 treatment in T-PLL cases (n=11) after 8h (blue, n=1 291), after 24h (yellow, n=4 801), after 8h upon 2ng/ml IL-6 IL-6 stimulation (green, n=1 543), and after 24h upon 2ng/ml IL-6 IL-6 stimulation (pink, n=4 218). **C** Volcano plot of DEGs upon 2.4µM JPX-1244 treatment and 2ng/ml IL-6 stimulation after 8h (top) and 24h (bottom), compared to untreated control upon 2ng/ml IL-6 stimulation. Genes significantly downregulated upon JPX-1244 treatment are marked in blue, and genes upregulated upon JPX-1244 treatment are colored in red, with a cut-off at $|log_2FC| \geqq 3$ and an FDR<0.05. **D** Expression of JAK/STAT genes in 11 T-PLL cases, one sample treated with 2.4µM JPX-1244 and one DMSO control of each case, both at the 24h timepoint, displayed in a heatmap. The JAK/STAT genes were adopted from the KEGG JAK/STAT gene set. The respective patient identification code (**Supplementary table 3**) is given in the top legend.

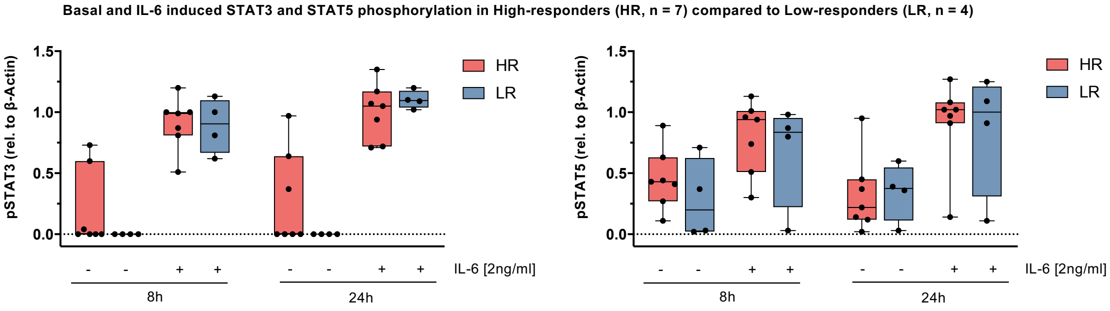

Supplementary Figure 5: JPX-1244 *ex-vivo* treatment responses are not associated with basal STAT3 or STAT5 phosphorylation

Densitometry of pSTAT3 (left) and pSTAT5 (right) signals in T-PLL patients, comparing JPX-1244 High-responders (HR, n = 7) and Low-responders (LR, n = 4), as defined by LD50s after 48h treatment in **Fig. 3B**. Box plots show the quantification of densitometry of pSTAT3 and pSTAT5 after 8h and 24h in culture, with and without 2ng/ml IL-6 stimulation, assessed by immunoblots and normalized to β-Actin (box plot with median as line, interquartile interval, and whiskers as minimum to maximum). No significant differences were observed in all comparisons of HR to LR in the respective conditions ($p>0.05$, two-tailed unpaired Student's t-test).

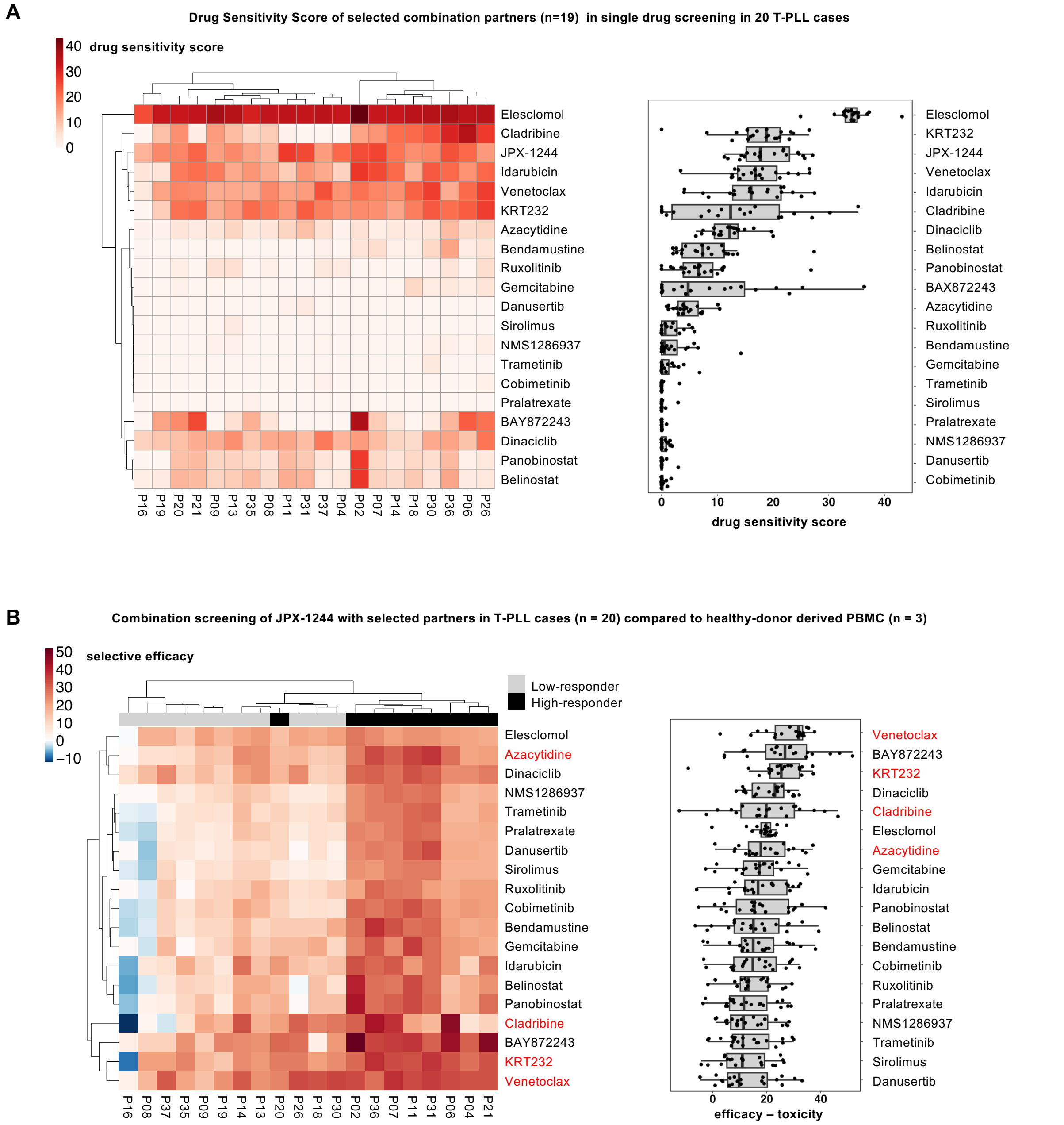

Supplementary Figure 6: Single-compound screening of JPX-1244 and 19 combination partners and selective efficacy of the combinations in primary T-PLL cells

A Drug sensitivity of JPX-1244 and 19 combination partners, as assessed by viability screening in 20 T-PLL cases upon 48h treatment with 6 increasing concentrations of each drug. Cell viability was assessed via CellTiter-Glo luminescent assay. Left: Heatmap showing color-coded drug sensitivity scores (DSS) of single-agent exposures to JPX-1244 and the 19 combination partners, presented per patient. Unsupervised hierarchical clustering of combination partners and T-PLL cases was performed. The respective patient ID is given in the bottom legend. The respective compound tested is given on

the right. Right: DSS of the 19 combination partners in 20 T-PLL cases displayed as box plot. The respective compound tested is given on the right. **B** Combination screening in 20 T-PLL cases and PBMC derived from 3 healthy donors. JPX-1244 was combined with 19 combination partners: KRT-232, belinostat, ruxolitinib, cladribine, bendamustine, trametinib, dinaciclib, azacytidine, elesclomol, danusertib, BAY872243, panobinostat, idarubicin, gemcitabine, sirolimus, pralatrexate, cobimetinib, venetoclax and NMS1286937. T-PLL samples and healthy controls were treated for 48h, with 6 increasing concentrations of each compound. Cell viability was assessed via CellTiter-Glo luminescent assay. Left: Heatmap showing color-coded selective efficacy of 19 combination therapies, presented per patient (red: selective efficacy >0, white: selective efficacy =0, blue: selective efficacy <0). Top legend displays the response status of each T-PLL patient towards JPX-1244 in previous single compound screenings (see **Fig. 3C**, grey: low-responder n=11, black: high-responder n=9). Unsupervised hierarchical clustering of both combination partners and T-PLL cases was performed. The respective patient ID is given in the bottom legend. The respective compound combined with JPX-1244 is given on the right. Right: Selective efficacy values of 19 combination strategies in 20 T-PLL cases compared to PBMC derived from healthy-donors (n=3) displayed in box plot. The respective compound combined with JPX-1244 is given on the right. For detailed information on the conditions of the performed drug testing, combination screening and calculation of DSS, selective efficacy and synergy, see **Supplementary Methods.**

References

1. Wahnschaffe L, Braun T, Timonen S, Giri AK, Schrader A, Wagle P, et al. JAK/STAT-Activating Genomic Alterations Are a Hallmark of T-PLL. Cancers. 2019 Nov 21;11(12).
2. Braun T, Glass M, Wahnschaffe L, Otte M, Mayer P, Franitza M, et al. Micro-RNA networks in T-cell prolymphocytic leukemia reflect T-cell activation and shape DNA damage response and survival pathways. Haematologica. 2022 Jan 1;107(1):187–200.
3. Staber PB, Herling M, Bellido M, Jacobsen ED, Davids MS, Kadia TM, et al. Consensus criteria for diagnosis, staging, and treatment response assessment of T-cell prolymphocytic leukemia. Blood. 2019 Oct 3;134(14):1132–43.
4. von Jan J, Timonen S, Braun T, Jiang Q, Ianevski A, Peng Y, et al. Optimizing drug combinations for T-PLL: restoring DNA damage and P53-mediated apoptotic responses. Blood. 2024 Oct 10;144(15):1595–610.
5. Braun T, Dechow A, Friedrich G, Seifert M, Stachelscheid J, Herling M. Advanced Pathogenetic Concepts in T-Cell Prolymphocytic Leukemia and Their Translational Impact. Front Oncol. 2021 Nov 19;11:775363.
6. Andersson EI, Pützer S, Yadav B, Dufva O, Khan S, He L, et al. Discovery of novel drug sensitivities in T-PLL by high-throughput ex vivo drug testing and mutation profiling. Leukemia. 2018 Mar;32(3):774–87.
7. Braun T, von Jan J, Wahnschaffe L, Herling M. Advances and Perspectives in the Treatment of T-PLL. Curr Hematol Malig Rep. 2020 Apr;15(2):113–24.
8. Ianevski A, Nader K, Driva K, Senkowski W, Bulanova D, Moyano-Galceran L, et al. Single-cell transcriptomes identify patient-tailored therapies for selective co-inhibition of cancer clones. Nat Commun. 2024 Oct 3;15(1):8579.
9. Ianevski A, Giri AK, Gautam P, Kononov A, Potdar S, Saarela J, et al. Prediction of drug combination effects with a minimal set of experiments. Nat Mach Intell. 2019 Dec 9;1(12):568–77.
10. He L, Tang J, Andersson EI, Timonen S, Koschmieder S, Wennerberg K, et al. Patient-Customized Drug Combination Prediction and Testing for T-cell Prolymphocytic Leukemia Patients. Cancer Res. 2018 May 1;78(9):2407–18.
11. Yadav B, Pemovska T, Szwajda A, Kulesskiy E, Kontro M, Karjalainen R, et al. Quantitative scoring of differential drug sensitivity for individually optimized anticancer therapies. Sci Rep. 2014 Jun 5;4(1):5193.
12. Chen Y, He L, Ianevski A, Ayuda-Durán P, Potdar S, Saarela J, et al. Robust scoring of selective drug responses for patient-tailored therapy selection. Nat Protoc. 2024 Jan;19(1):60–82.
13. Ianevski A, Giri AK, Aittokallio T. SynergyFinder 3.0: an interactive analysis and consensus interpretation of multi-drug synergies across multiple samples. Nucleic Acids Res. 2022 Jul 5;50(W1):W739–43.
14. Ianevski A, Timonen S, Kononov A, Aittokallio T, Giri AK. SynToxProfiler: An interactive analysis of drug combination synergy, toxicity and efficacy. PLoS Comput Biol. 2020 Feb;16(2):e1007604.
15. Langmead B, Salzberg SL. Fast gapped-read alignment with Bowtie 2. Nat Methods. 2012 Mar 4;9(4):357–9.
16. Danecek P, Bonfield JK, Liddle J, Marshall J, Ohan V, Pollard MO, et al. Twelve years of SAMtools and BCFtools. GigaScience. 2021 Feb 16;10(2):giab008.
17. Kumar A, Kankainen M, Parsons A, Kallioniemi O, Mattila P, Heckman CA. The impact of RNA sequence library construction protocols on transcriptomic profiling of leukemia. BMC Genomics. 2017 Dec;18(1):629.
18. Wang K, Li M, Hakonarson H. ANNOVAR: functional annotation of genetic variants from high-throughput sequencing data. Nucleic Acids Res. 2010 Sep;38(16):e164.
19. Mayakonda A, Lin DC, Assenov Y, Plass C, Koeffler HP. Maftools: efficient and comprehensive analysis of somatic variants in cancer. Genome Res. 2018 Nov;28(11):1747–56.
20. Love MI, Huber W, Anders S. Moderated estimation of fold change and dispersion for RNA-seq data with DESeq2. Genome Biol. 2014 Dec 5;15(12):550.
21. Yu G, Wang LG, Han Y, He QY. clusterProfiler: an R package for comparing biological themes among gene clusters. Omics J Integr Biol. 2012 May;16(5):284–7.
